# Soft Wetting Ridge Rotation in Sessile Droplets and Capillary Bridges

**DOI:** 10.1021/acs.langmuir.4c04667

**Published:** 2025-02-03

**Authors:** Bo Xue Zheng, Tak Shing Chan

**Affiliations:** Mechanics Division, Department of Mathematics, University of Oslo, 0316 Oslo, Norway

## Abstract

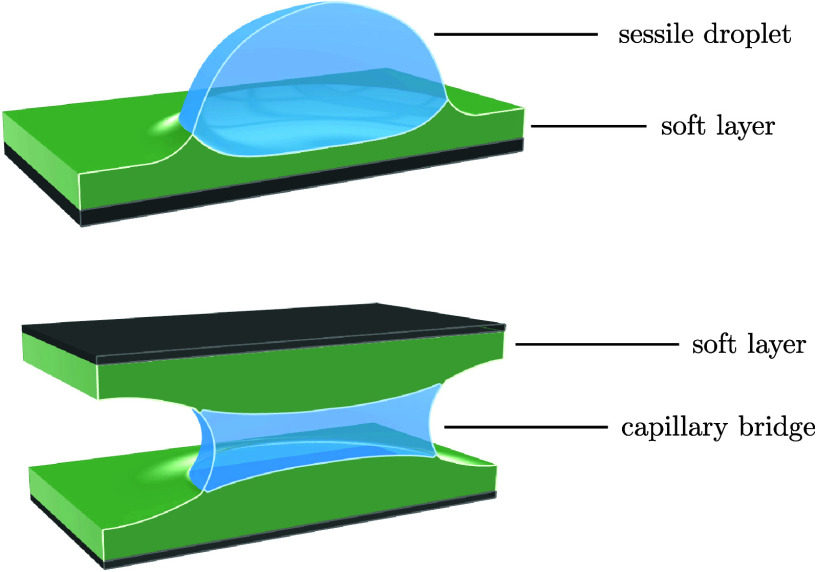

We investigate the deformation of soft solid layers in the presence of sessile droplets or capillary bridges. Unlike models that assume Young’s law governs the contact angle, we incorporate the surface tension balance at the contact line to analyze the rotation of the wetting ridge and the corresponding change in the contact angle. Our findings reveal that the rotation direction of the wetting ridge aligns with the sign of the Laplace pressure. Interestingly, although a softer solid layer typically decreases the contact angle for sessile droplets, a negative Laplace pressure in a hydrophilic capillary bridge pulls the solid–liquid interface, leading to an increased contact angle. A hydrophilic capillary bridge would be expected to move from thicker regions of a soft layer to thinner areas, exhibiting behavior opposite that of a sessile droplet. The interplay between soft layer deformation and contact angle modulation provides valuable insights into controlling droplet motion through elastocapillarity.

## Introduction

Manipulating surface wettability to control droplet morphology and movement^1^ has broad applications in industrial processes and is widely observed in natural phenomena. Over the past decades, extensive research has focused on droplets in contact with substrates coated with soft material layers^2−17^ such as gels^18,19^ and elastomers. Studies have shown that manipulating the softness of the layer can provide an alternative way to control the motion of droplets.^20−27^ Style et al.^20^ demonstrated experimentally that a sessile droplet moves spontaneously from thinner to thicker regions of a soft layer. This phenomenon, known as durotaxis, originally describes the process that biological cells move along gradients in the stiffness of a substrate. The movement of the droplet is explained in terms of a decrease in the contact angle with the thickness of the elastic layer. The thickness gradient creates a variation in the contact angle along the contact line, which drives the motion.

**Figure 1:**
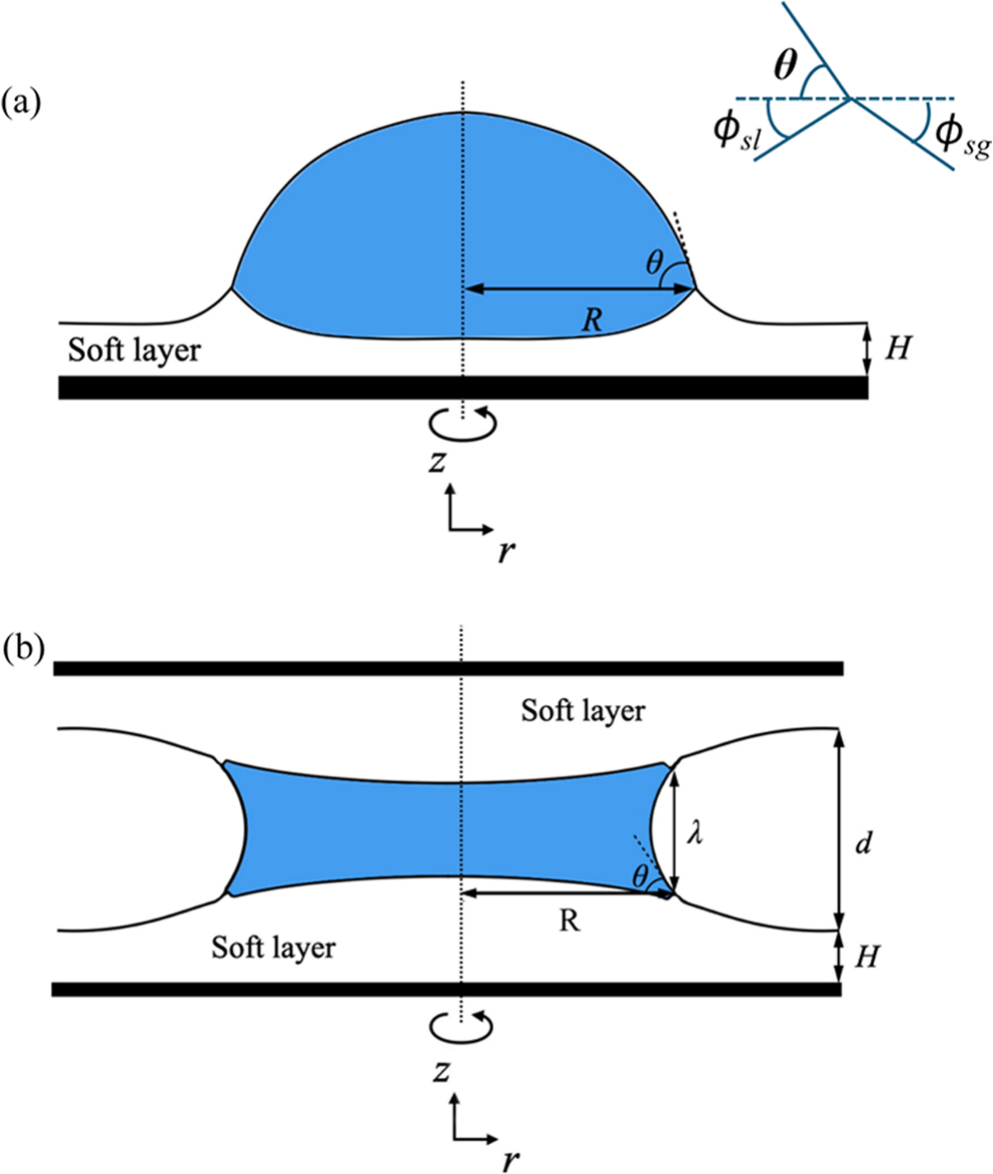
Schematic diagrams of an axisymmetric droplet in contact with one single substrate in panel (a), i.e., a sessile droplet; and two parallel substrates in panel (b), i.e., a capillary bridge. The substrates consist of rigid plates coated with a soft elastic layer of thickness *H*. The soft layer is deformed by the droplet to form a wetting ridge. At the contact line position, the sessile droplet or the capillary bridge makes an equilibrium contact angle θ with the substrates. The angles of the interfaces, θ, ϕ_sl_, and ϕ_sg_, follow the balance of surface tensions, also known as Neumann’s triangle.

For droplets in contact with a rigid substrate, the contact angle θ is often assumed to follow Young’s law: i.e., θ = θ_*Y*_, with the Young’s angle θ_*Y*_ determined by
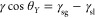
1where, γ, γ_sg_, and γ_sl_ are respectively the liquid–gas, solid–gas, and solid–liquid surface tensions. However, when a droplet contacts a soft substrate, see Figure 1, the soft layer deforms to form a ridge-like shape due to a pulling capillary force at the contact lines.^12,14,28^ Minimizing the free energy,^29^ which includes both the surface energies and the elastic energy, reveals that the angles between the interfaces at the contact line follow the balance of the surface tensions,^7,29^ also known as Neumann’s triangle. This condition leaves one degree of freedom for determining the angles, as the surface tension vectors can be rotated by the same angle without violating the force balance. As a result, the angles are fixed only when considered together with deformation away from the contact line.

Studies of sessile droplet with θ_*Y*_ = 90°^30,31^ show that the contact angle decreases significantly from the Young’s angle when increasing the softness parameter *S* for *S* ≳ 1, where *S* ≡ γ/*ER* is defined as the ratio of two length scales: the elastocapillary length γ/*E* and the contact radius of the droplet *R*. Here, *E* is the Young’s modulus. In the limit of very soft case, i.e., *S* ≫ 1, droplets appear as a lens floating on a liquid bath for which surface tensions completely dominate over elastic stresses.^30,31^

Despite remarkable findings, most studies on soft wetting are restricted to sessile droplets and θ_*Y*_ = 90°. It remains unclear how the contact angle is modified in other geometrical confinements. Even on planar surfaces, a droplet in contact with two parallel plates forms a capillary bridge,^32−40^ which has a significantly different shape from a sessile droplet. Studies of the capillary bridge between two soft layers are rather limited. It has been shown that the soft layer is drawn toward the bridge by the pulling contact line force and the negative Laplace pressure.^41,42^ In those studies, the authors either consider only the completely wetting case,^41^ i.e., θ_*Y*_ = 0°, or assume a smooth solid interface at the contact line position and a local Young’s law.^42^

In this article, we impose the condition of the surface tension balance at the contact line and examine both hydrophilic and hydrophobic surfaces. We unravel the morphology of the wetting ridge for droplets in contact with planar soft layers in two common cases: sessile droplets and capillary bridges. Our results demonstrate how the contact angle depends on the geometric parameters and the softness of the solid layers.

## Methods

We consider substrates that consist of a rigid plate coated with a soft elastic layer of uniform thickness *H* in an undeformed state. A droplet of volume *V* and density ρ is placed in contact with (1) one single substrate to form a sessile droplet and (2) two parallel substrates to form a capillary bridge, as shown in the schematics in Figure 1a,b respectively. The schematics also illustrate the deformation of the soft layers due to the droplet/bridge Laplace pressure and the pulling capillary force at the contact lines. For the bridge case, the gap separation between the two undeformed soft layers is *d*, which is assumed to be much smaller than the radius of the contact line *R*, i.e., *d* ≪ *R*. The distance between the contact lines at the top and the bottom is denoted as λ. Due to the axisymmetry of the problem, we will use the cylindrical coordinate system (*r*, *z*, ϕ). The droplet/bridge makes a contact angle of θ with the soft layers at the contact line positions *r* = *R*. Note that the contact angle is measured with respect to the plane parallel to the undeformed soft layers. The contact angle is an unknown variable that has to be determined together with the solution of the solid interface deformation. Instead of using Young’s law (eq 1), we use the condition of balance of surface tensions at the contact line, which will be described in detail in the later part of this section. We denote the displacement in the soft layers as ***U***(*r*, *z*) = *U*_*r*_***r̂*** + *U*_*z*_***ẑ*** + *U*_ϕ_**ϕ̂**. Employing linear elasticity, the relation between the stress tensor **σ** and the strain tensor **ϵ** = [∇**U** + (∇**U**)^**T**^]/2 is given by
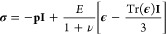
2where, *p* is the pressure in the elastic layer, ν is the Poisson ratio, **I** is the identity tensor, and Tr represents taking the trace of a tensor. In Supporting Information 1, we give the relations between the components of the tensors in cylindrical coordinates. Denoting the gravitational acceleration as *g*, we consider the Bond number *Bo* ≡ *ρgV*^2/3^/γ ≪ 1, and thus, the effect of gravity on the droplet/bridge shape is negligible. We consider static states, the deformation in the soft layer is governed by the force balance equation:
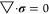
3

For the bridge case, the deformations of the top and the bottom soft layers are the same, and hence we only focus on the bottom one. We consider a rectangular domain of computation with *r* = [0, *L*] and *z* = [0, *H*], which represents the undeformed shape (reference state) of the soft layer. The boundary conditions are as follows. At a distance *r* = *L* far from the droplet/bridge, we impose the condition ***U***(*r* = *L*, *z*) = 0. At *r* = 0, we have *U*_*r*_ = 0 and ∂*U*_*z*_/∂*r* = 0 due to symmetry. At the interface where the soft layer is in contact with the rigid substrate, i.e., *z* = 0, the soft material is undeformed, so we have ***U***(*r*, *z* = 0) = 0.

At the boundary where the soft layer is in contact with the fluids, i.e., *z* = *H*, we impose the force balance condition. Denoting a force acting on the interface over a radial length *δr* as ***f****δr*, we introduce all of the traction terms ***f*** in the following. First, the capillary traction ***f***^*l*^ due to the liquid–gas surface tension is pulling the soft layer at the contact lines. The capillary traction is expressed as
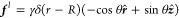
4where, δ(*r* – *R*) is the Dirac delta function.^11^ Second, we give the expression for elastic traction ***f***^el^. As our model employs linear elasticity, it is natural to write the elastic traction ***f***^el^ with respect to the undeformed configuration, given by
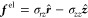
5One might wonder about the validity of using linear elasticity at the contact line region, where the deformed interfacial slope is generally not small. However, as surface tensions dominate over elastic stresses at the contact line,^7,15^ the inaccuracies in the elastic stress calculation are expected to be less significant. Thus, the linear elasticity remains a reasonable approximation for our computations.

Third, we provide the expression for the traction generated by the solid surface tension γ_*s*_. Note that this traction is computed based on the deformed interface. The traction is given by^29^
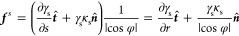
6where, ***t̂*** ≡ cos φ***r̂*** + sin φ***ẑ*** and ***n̂*** ≡ – sin φ***r̂*** + cos φ***ẑ*** are respectively the tangential unit vector and the normal unit vector of the deformed soft layer interface, and  is the local arc length. The corresponding local angle φ is computed from the slope of the deformed interface as
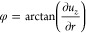
7with *u*_*z*_ ≡ *U*_*z*_(*z* = *H*). Due to the change in the length of the deformed interface relative to the undeformed shape, a factor of 1/|cos φ| is introduced to account for this variation. The curvature of the deformed soft layer interface κ_*s*_ is given by
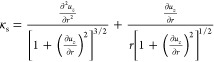
8

The value of solid surface tension γ_*s*_ exhibits a discontinuity when crossing from the solid–liquid interface to the solid–gas interface, that is γ_s_ = γ_sl_ + (γ_sg_ – γ_sl_) *H*_s_(*r* – *R*) = γ_sl_ + γ cos θ_*Y*_*H*_s_(*r* – *R*), where *H*_s_(*r* – *R*) is the Heaviside step function. For soft solids, we also have to distinguish between surface energy and surface stress due to Shutterworth effect.^43−45^ In this study, we neglect this effect and assume that the tension force at the solid interface is independent of the stretching force of the solid.

Fourthly, the Laplace pressure generated inside the droplet/bridge is pressing or pulling the solid–liquid interface. The traction ***f***^*La*^ due to the Laplace pressure is given by
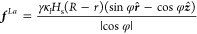
9where, the curvature of the liquid–air interface is
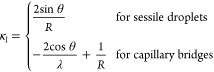
10Remember that for the bridge case, we assume that *R* ≫ λ. We can see that κ_*l*_ is positive for both hydrophilic and hydrophobic surfaces in the sessile droplet case. For the bridge case, κ_*l*_ is negative for hydrophilic surfaces when cos θ > λ/2*R*.

Balancing all of the tractions, we have the following boundary condition at *z* = *H*,
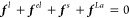
11To determine the contact angle θ, we impose the condition that surface tensions balance each other at the contact line, which means ***f***^*l*^ and ***f***^*s*^ dominate over ***f***^*La*^ and ***f***^*el*^. Denoting the angles of the solid–liquid and solid–gas interfaces respectively as ϕ_*sl*_ and ϕ_*sg*_, see Figure 1, balancing the surface tensions gives
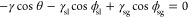
12and
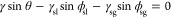
13The values of ϕ_sl_ and ϕ_sg_ are determined from the profiles of the deformed solid interface. Numerically to find the solution of the contact angle θ, we start with a trial value θ = θ_*Y*_ and compute the profile of the deformed solid interface to obtain ϕ_sl_ and ϕ_sg_ for the trial value of θ. We iterate the trial value of θ until the conditions 12 and 13 are fulfilled.

In this study, we consider that the soft material is incompressible, which means that ν = 0.5. The incompressibility condition implies
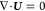
14

Next, we nondimensionalize the variables as the following. We introduce a length scale *l* to rescale all of the lengths and displacements. We take *l* = *R* for the sessile droplet case, and *l* = *d* for the capillary bridge case. The stresses are rescaled by *E*. The dimensionless lengths, displacements, and stresses are *r̃* = *r/l*, *z̃* = *z/l*, *H̃* = *H/l*, *Ũ*_*r*_ = *U*_*r*_/*l*, *Ũ*_*z*_ = *U*_*z*_/*l*, *p̃* = 3*p/E*, and **σ̃** = **σ**/*E*. See Supporting Information 2 for the dimensionless governing equations and boundary conditions. Moreover, to reduce the number of control parameters, we consider only situations in which γ_sl_/γ = 1. We end up with the following dimensionless control parameters:
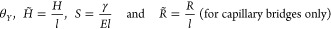
15The dimensionless governing equations with boundary conditions (shown in Supporting Information 2) are solved using the finite element method for which the details are given in Supporting Information 3.

## Results and Discussion

### Validation: Sessile Droplets with θ_*Y*_ = 90° on a Thick Soft Layer

**Figure 2:**
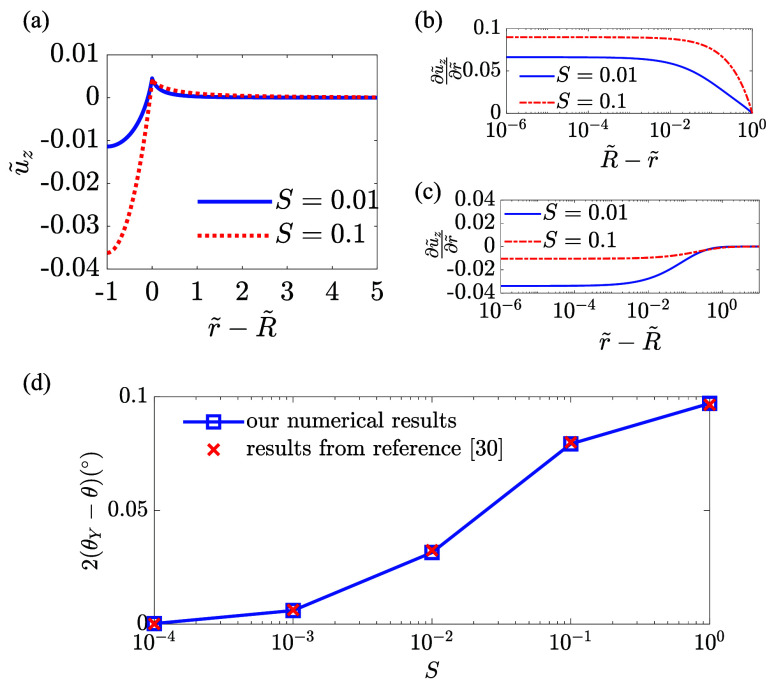
Sessile droplet case with θ_*Y*_ = 90° and *H̃* = 10. (a) Rescaled displacement *ũ*_*z*_ as a function of *r̃* – *R̃*. (b, c) Slope  as a function of *R̃* – *r̃* in panel (b) for the solid–liquid interface and *r̃* – *R̃* in panel (c) for the solid–gas interface. (d) Change of contact angle 2(θ_*Y*_ – θ) as a function of the softness parameter *S*. Our numerical results are compared with results in the ref (30). Reproduced with permission from reference 30. Copyright 2014 Cambridge University Press.

We validate our approach by comparing the contact angle change with the results in the ref (30) where the authors study a sessile droplet with θ_*Y*_ = 90° on a semi-infinite thick soft substrate. We compute the soft layer deformation for a sessile droplet on a thick soft layer with *H̃* = 10 and θ_*Y*_ = 90°. Note that Young’s angle 90° implies γ_sl_ = γ_sg_. Figure 2a depicts the rescaled displacement *ũ*_*z*_ ≡ *Ũ*_*z*_(*z̃* = *H̃*) as a function of *r̃* – *R̃* for two different values of softness parameter, i.e., *S* = 0.01 and 0.1. We can see the solid interface deforms upward to form a wetting ridge around the contact line position, i.e., *r̃* – *R̃* = 0, due to the pulling capillary force. Notably, although γ_sl_ = γ_sg_, the interface deforms differently on the solid–liquid side and on the solid–gas side. The solid–liquid interface forms a pronounced dimple resulted from the pressing by the Laplace pressure. The dimple is deeper for the softer layer at *S* = 0.1. Next, to measure the angles of the interfaces at the contact line, we compute the slope of the solid interface which is shown in Figure 2b,c as a function of *R̃* – *r̃* (or *r̃* – *R̃*) in log-scale, respectively, for the solid–liquid side and the solid–gas side. We see when decreasing |*R̃* – *r̃*|, the slope reaches a plateau which indicates the range of interface where surface tensions dominate over elastic stresses.^16^ The angles ϕ_sl_ and ϕ_sg_ are determined from the slope at the plateau. Figure 2d shows the derivations of θ from the Young’s angle θ_*Y*_ as a function of the softness parameter *S*. The results obtained by our method show perfect agreement with those in the ref (30).

### Deformation of the Soft Layer for the Sessile Droplet Case

**Figure 3:**
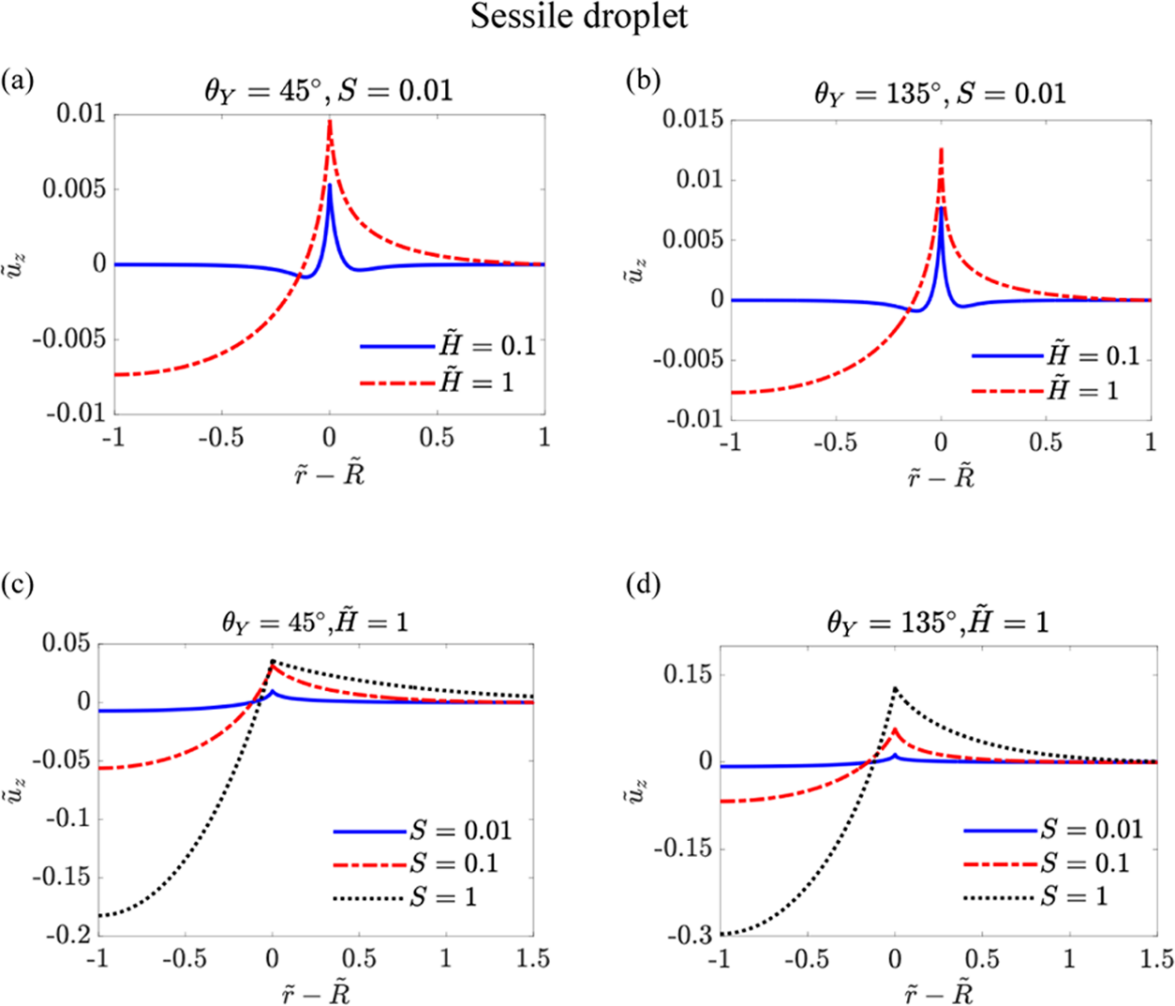
Rescaled displacement *ũ*_*z*_ as a function of *r̃* – *R̃* for the sessile droplet case. In (a) θ_*Y*_ = 45° and *S* = 0.01, with different rescaled thickness of the soft layer, i.e., *H̃* = 0.1, 1; (b) θ_*Y*_ = 135° and *S* = 0.01 with different rescaled thickness of the soft layer, i.e., *H̃* = 0.1, 1; (c) θ_*Y*_ = 45° and *H̃* = 1 with different softness parameter of the layer *S*, and (d) θ_*Y*_ = 135° and *H̃* = 1 with different softness parameter of the layer.

We examine the sessile droplet case for a hydrophilic surface of θ_*Y*_ = 45° and a hydrophobic surface of θ_*Y*_ = 135°. Figure 3 shows the rescaled displacement *ũ*_*z*_ as a function of *r̃* – *R̃*. In Figure 3a,b, we fix the value of *S* = 0.01 and compare the profiles for *H̃* = 0.1 and *H̃* = 1. In Figure 3c,d, we compare the profiles for *S* = 0.01, *S* = 0.1, and *S* = 1 with a fixed value of *H̃* = 1. For cases of *H̃* = 0.1, which means that the soft layer thickness is smaller than the droplet contact radius, we find that a small dimple is formed on both the solid–liquid and solid–gas sides at *r̃* – *R̃* ≈ *H̃*. However, for *H̃* = 1, a dimple extends across the entire region of 0 < *r̃* < *R̃*. The asymmetry in deformation between the solid–liquid and solid–gas sides becomes more pronounced with larger values of *H̃* or *S*. Notably, the deformation characteristics are similar for θ_*Y*_ = 45° and θ_*Y*_ = 135°.

### Deformation of the Soft Layer for the Capillary Bridge Case

**Figure 4:**
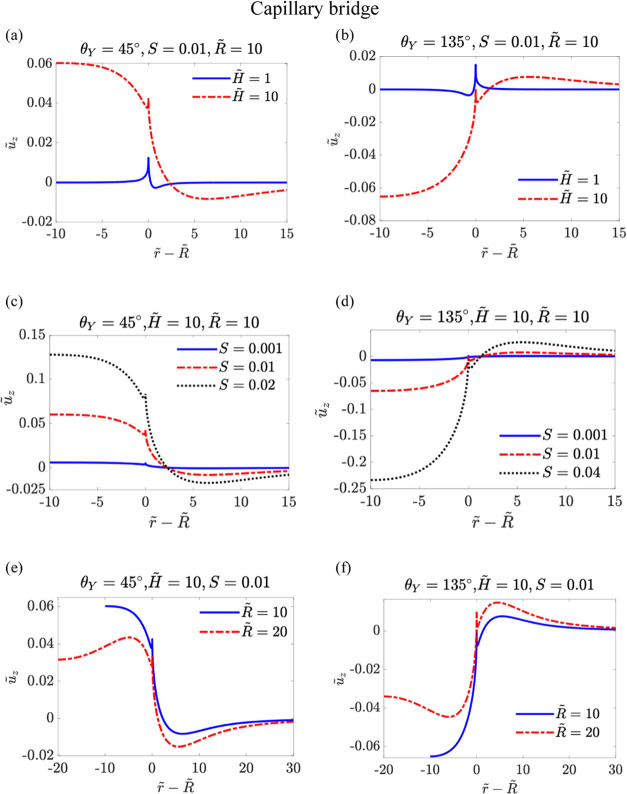
Rescaled displacement *ũ*_*z*_ as a function of *r̃* – *R̃* for the capillary bridge case. In (a) θ_*Y*_ = 45°, *S* = 0.01 and *R̃* = 10 with different rescaled thickness of the substrate i.e., *H̃* = 1, 10; (b) θ_*Y*_ = 135°, *S* = 0.01 and *R̃* = 10, with different rescaled thickness of the substrate i.e., *H̃* = 1, 10; (c) θ_*Y*_ = 45°, *H̃* = 1 and *R̃* = 10, with different softness parameter of the substrate *S*; (d) θ_*Y*_ = 135°, *H̃* = 1, *R̃* = 10, with different softness parameter of the substrate *S*; (e) θ_*Y*_ = 45°, *H̃* = 1, *S* = 0.01, with different rescaled radius of the capillary bridge i.e., *R̃* = 10, 20; (f) θ_*Y*_ = 135°, *H̃* = 1, *S* = 0.01, with different rescaled radius of the capillary bridge i.e., *R̃* = 10, 20.

Similarly, we examine the capillary bridge case for θ_*Y*_ = 45° and θ_*Y*_ = 135°. Remind that all of the dimensionless lengths here are rescaled by the gap separation of the two soft layers rather than by the droplet contact radius. In Figure 4, we plot the rescaled displacement *ũ*_*z*_ as a function of *r̃* – *R̃*. We see that a wetting ridge with a sharp tip forms at the contact line region, contrasting with results from previous studies of soft wetting by capillary bridges.^41,42^ Additionally, as shown in Figure 4a,b, increasing *H̃* shifts the maximum value of *ũ*_*z*_ (denoted as *ũ*_*zm*_) from the contact line position to the solid–liquid side for θ_*Y*_ = 45° and to the solid–gas side for θ_*Y*_ = 135°. This shift suggests that when the top and bottom soft layers make contact, i.e., *ũ*_*zm*_ = 0.5, the contact point is not at the contact line position.^42^

Compared with the sessile droplet case, we observed several key differences. For θ_*Y*_ = 45°, shown in Figure 4a,c,e, the Laplace pressure pulls the solid–liquid surface, resulting in a positive displacement for *r̃* – *R̃* < 0. For θ_*Y*_ = 135° shown in Figure 4b,d,f, the Laplace pressure presses the solid–liquid interface to form a pronounced dimple, which is similar to that seen in the sessile droplet case. However, a crest (maximum *ũ*_*z*_) can also appear on the solid–gas side, which is not observed for the sessile droplet case. We hypothesize that this results from a stronger Laplace pressure effect in the capillary bridge compared to the sessile droplet relative to the pulling capillary force at the contact line. As with the sessile droplet case, the asymmetry in deformation between the solid–liquid and solid–gas sides is more pronounced with larger *H̃* or *S*. In Figure 4e,f, we also observe that varying *R̃* alters the deformation features, specifically shifting the positions of the maximum and minimum values of *ũ*_*z*_.

### Wetting Ridge Rotation and the Change of Contact Angle

**Figure 5:**
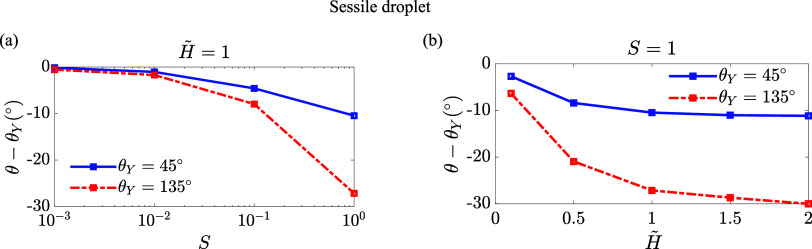
Contact angle variation θ – θ_*Y*_ as a function of the softness parameter *S* in panel (a) and the rescaled soft layer thickness *H̃* in panel (b) for the sessile droplet case. The blue solid line and red dashed-dotted lines represent θ_*Y*_ = 45° and θ_*Y*_ = 135° cases, respectively.

**Figure 6:**
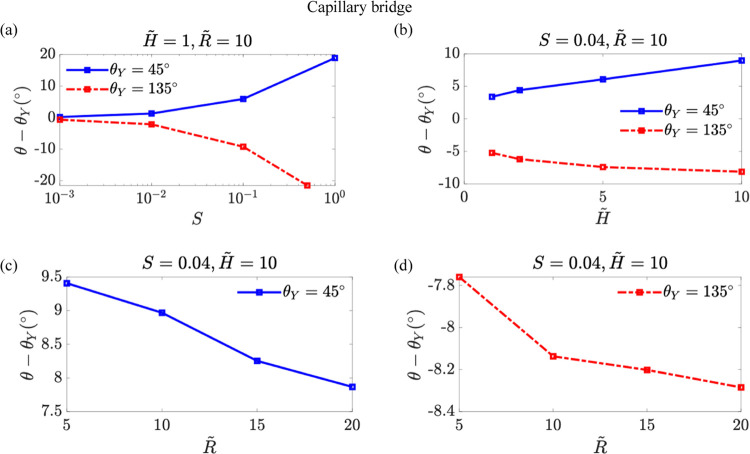
Contact angle variation θ – θ_*Y*_ as a function of the softness parameter *S* in panel (a), the rescaled soft layer thickness *H̃* in panel (b) and the rescaled contact radius *R̃* in panels (c) and (d) for the capillary bridge case. The blue solid line and the red dashed-dot lines represent θ_*Y*_ = 45° and θ_*Y*_ = 135° cases, respectively.

We present how the contact angle variation, characterized by θ – θ_*Y*_, changes with the control parameters for the sessile droplet case in Figure 5 and the capillary bridge case in Figure 6. When the softness parameter *S* ≪ 1, the contact angle remains equal to the Young’s angle as shown in Figures 5a and 6a. In the sessile droplet case (Figure 5), for both θ_*Y*_ = 45° and θ_*Y*_ = 135°, the contact angle θ is smaller than the Young’s angle, indicating a counterclockwise rotation of the wetting ridge. The magnitude of this rotation increases with both *S* and *H̃*. In contrast, for the capillary bridge case (Figure 6), the contact angle θ is smaller than the Young’s angle for θ_*Y*_ = 135° but larger for θ_*Y*_ = 45°. This means the wetting ridge rotates clockwise for θ_*Y*_ = 45°. The increase in θ from the Young’s angle at θ_*Y*_ = 45° becomes more pronounced when enhancing *S* or *H̃*, or with a decrease in *R̃*.

**Figure 7:**
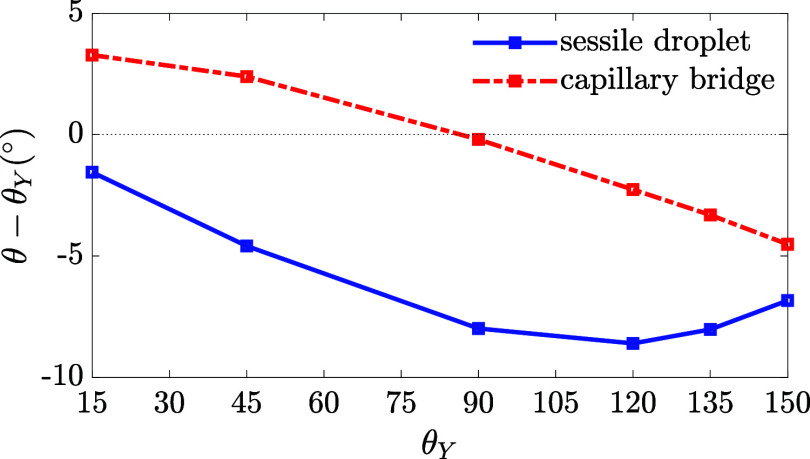
Contact angle variation θ – θ_*Y*_ as a function of Young’s angle θ_*Y*_. The blue line is for the sessile droplet case. Parameters: *S* = 0.1 and *H̃* = 1. The red line is for the capillary bridge case. Parameters: *S* = 0.01, *H̃* = 10, and *R̃* = 10.

The direction of the wetting ridge rotation aligns with the sign of the Laplace pressure, *γκ*_*l*_, with κ_*l*_ given by eq 10. For the sessile droplet case and the capillary bridge with θ_*Y*_ = 135°, a positive Laplace pressure presses the solid–liquid interface, causing the ridge to rotate counterclockwise and reducing θ. Conversely, in the capillary bridge with θ_*Y*_ = 45°, a negative Laplace pressure pulls the solid–liquid interface, leading to an increase in the θ. To compare the contact angle change relative to Young’s angle between the sessile droplet and capillary bridge cases, we plot θ – θ_*Y*_ for various values of θ_*Y*_ in Figure 7. Our results show that θ – θ_*Y*_ is always negative in the sessile droplet case. The magnitude of rotation decreases when θ_*Y*_ approaches 0° or 180°, where the Laplace pressure becomes negligible. Consequently, a maximum rotation occurs at an intermediate Young’s angle. In the capillary bridge case, the wetting ridge rotates counterclockwise for hydrophilic surfaces with θ_*Y*_ ≲ 85° and clockwise for surfaces with θ_*Y*_ ≳ 85°. Note that Laplace pressure vanishes when θ is slightly smaller than 90°, see eq 10.

In a numerical study by Bueno et al.^22^ employing a nonlinear elasticity model with a diffuse interface, it is shown that sessile droplets on hydrophilic surfaces migrate toward thicker regions of a soft layer, consistent with experimental observations by Style et al.^20^ Remarkably, on hydrophobic surfaces, the droplets move in an opposite direction, namely, from thicker regions to thinner regions. The study further reveals that on such surfaces, the contact angle becomes larger when the layer is softer or thicker. The authors point out that although Laplace pressure rotates the ridge toward the droplet side, resulting in a reduced contact angle, the capillary force at the contact line tends to rotate the ridge in the opposite direction when the surface is hydrophobic. This effect dominates when θ_*Y*_ is above a critical value. In contrast, our results show that the wetting ridge rotates in the same direction for all values of θ_*Y*_ as illustrated in Figure 7 for the sessile droplet case. Interestingly, we observe that the maximum magnitude of the contact angle variation occurs when the surface is hydrophobic. Given the differences in model assumptions, such as nonlinearity and boundary conditions at the contact line, further investigation is needed to reconcile these discrepancies.

**Figure 8:**
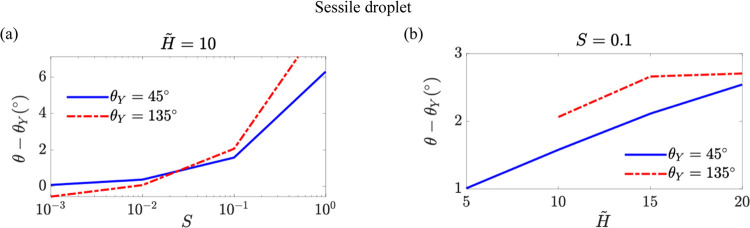
Contact angle variation θ – θ_*Y*_ as a function of the softness parameter *S* in panel (a) and the rescaled thickness of the soft layer *H̃* in panel (b) for the sessile droplet case when the Laplace pressure term ***f***^*La*^ in eq 11 is removed from our computation. The blue solid line and the red dashed-dotted lines respectively represent the results for the hydrophilic surface of θ_*Y*_ = 45° and hydrophobic surface of θ_*Y*_ = 135°.

To further explore this, we examine a specific situation in which the Laplace pressure term ***f***^*La*^ in eq 11 is removed from our computation, leaving only the capillary pulling force at the contact line acting on the soft layer. In Figure 8, we show the resulting contact angle variation for the sessile droplet case. We find that the capillary pulling force alone rotates the ridge such that the contact angle is enhanced as *S* or *H̃* increases for both the hydrophilic surface of θ_*Y*_ = 45° and the hydrophobic surface of θ_*Y*_ = 135°. This rotation direction is opposite to that induced by the Laplace pressure. Nevertheless, as our complete analysis indicates, the direction of ridge rotation is still governed by the sign of the Laplace pressure. Future experimental studies will be crucial for clarifying the physical mechanisms underlying these behaviors.

## Conclusions

Contrary to models that assume Young’s relation for partially wetting scenarios^10^ or limit the analysis to θ_*Y*_ = 90°,^30,31,42^ our approach incorporates a comprehensive surface tension balance condition at the contact line, enabling it to account for a broader range of contact angle and softness of the layer. We unravel the morphology of the wetting ridge for both sessile droplets and capillary bridges. The direction of the wetting ridge rotation critically depends on the sign of the Laplace pressure. For example, for the hydrophilic capillary bridge case, a negative Laplace pressure pulls the solid–liquid interface and the contact angle is enhanced from the Young’s angle. Assuming that a droplet migrates from regions of higher to lower surface energy, a hydrophilic capillary bridge is expected to move from thicker regions of a soft layer toward thinner areas, in contrast to the behavior observed for a sessile droplet.

The complex interplay between soft layer deformation and droplet contact angle remains far from fully understood, particularly in confined geometries beyond the classic sessile droplet case. Extensive theoretical and experimental investigations are needed to deepen our understanding in this area, providing valuable insights into controlling droplet motion through elastocapillarity.

## Supporting Information Available

S1: Relations between the components of the stress tensor and the displacements in cylindrical coordinates. S2: The dimensionless governing equations and boundary conditions. S3: Finite element method. (PDF)
Additional data. (ZIP)
