# Supplementary material for: Soft Wetting Ridge Rotation in Sessile Droplets and Capillary Bridges
Source: Langmuir. 2025 Feb 3;41(6):4146–53. doi: 10.1021/acs.langmuir.4c04667 (PMC11841037; doi:10.1021/acs.langmuir.4c04667)
Supplement: Supplementary file 2 — la4c04667_si_002.zip [file la4c04667_si_002.zip › SupportingInformation/untitled folder/SupportingInformation.pdf]

Soft wetting ridge rotation in sessile droplets and capillary bridges (Supporting information)

Bo Xue Zheng and Tak Shing Chan*

Mechanics Division, Department of Mathematics, University of Oslo, 0316 Oslo, Norway

E-mail: taksc@uio.no

SI1: Relations between the components of the stress tensor and the displacements in cylindrical coordinates

$$\sigma_{rr} = -p + \frac{E}{(1+\nu)}\left(\frac{\partial U_r}{\partial r} - \frac{1}{3}\nabla \cdot \boldsymbol{U}\right), \tag{1}$$

$$\sigma_{zz} = -p + \frac{E}{(1+\nu)}\left(\frac{\partial U_z}{\partial z} - \frac{1}{3}\nabla \cdot \boldsymbol{U}\right), \tag{2}$$

$$\sigma_{\phi\phi} = -p + \frac{E}{(1+\nu)}\left(\frac{U_r}{r} - \frac{1}{3}\nabla \cdot \boldsymbol{U}\right), \tag{3}$$

$$\sigma_{rz} = \frac{E}{(1+\nu)}\left(\frac{\partial U_r}{\partial z} + \frac{\partial U_z}{\partial r}\right), \tag{4}$$

where the isotropic part of the stress tensor (or the pressure)

$$p = -\frac{E}{3(1-2\nu)}\nabla \cdot \boldsymbol{U}. \tag{5}$$

SI2: The dimensionless governing equations and boundary conditions

For the elastic deformation, the dimensionless form of $\bigtriangledown \cdot \boldsymbol{\sigma} = 0$ is

$$\tilde{\nabla}^2 \tilde{U}_r - \frac{\tilde{U}_r}{\tilde{r}^2} - \frac{\partial \tilde{p}}{\partial \tilde{r}} = 0 \tag{6}$$

in r-direction and

$$\tilde{\nabla}^2 \tilde{U}_z - \frac{\partial \tilde{p}}{\partial \tilde{z}} = 0 \tag{7}$$

in z-direction. The dimensionless form of the incompressibility condition (eq. **??**) is

$$\tilde{\nabla} \cdot \tilde{\boldsymbol{U}} = 0. \tag{8}$$

The boundary conditions far away from the droplet at $\tilde{r} = \tilde{L}$ and at the soft/rigid solid interface respectively are

$$\tilde{\boldsymbol{U}}(\tilde{r} = \tilde{L}, \tilde{z}) = 0 \tag{9}$$

and

$$\tilde{\boldsymbol{U}}(\tilde{r}, \tilde{z} = 0) = 0. \tag{10}$$

At $\tilde{r}=0$, the symmetry property gives

$$\tilde{U}_r(\tilde{r}=0,\tilde{z})=0, \tag{11}$$

and

$$\frac{\partial \tilde{U}_z}{\partial \tilde{r}}(\tilde{r}=0,\tilde{z})=0. \tag{12}$$

At the soft solid/fluid interface $\tilde{z}=\tilde{H}$, the force balance condition (eq. **??**) for the r-components and z-components respectively gives

$$\begin{aligned}\frac{\tilde{\sigma}_{rz}}{S}+\tilde{\kappa}_l H_s(\tilde{R}-\tilde{r})\frac{\sin\varphi}{|\cos\varphi|}-\cos\theta\,\delta(\tilde{r}-\tilde{R})\\ -\tilde{\gamma}_s\tilde{\kappa}_s\frac{\sin\varphi}{|\cos\varphi|}\\ +\frac{\partial\tilde{\gamma}_s}{\partial\tilde{r}}\cos\varphi=0.\end{aligned} \tag{13}$$

and

$$\begin{aligned}-\frac{\tilde{\sigma}_{zz}}{S}-\tilde{\kappa}_l H_s(\tilde{R}-\tilde{r})\,\mathrm{sgn}(\cos\varphi)+\sin\theta\,\delta(\tilde{r}-\tilde{R})\\ +\tilde{\gamma}_s\tilde{\kappa}_s\,\mathrm{sgn}(\cos\varphi)\\ +\frac{\partial\tilde{\gamma}_s}{\partial\tilde{r}}\sin\varphi=0\end{aligned} \tag{14}$$

where $\tilde{\kappa}_l=\kappa_l l$, $\tilde{\kappa}_s=\kappa_s l$, $\tilde{\gamma}_s=\frac{\gamma_s}{\gamma}$ and $\frac{\partial\tilde{\gamma}_s}{\partial\tilde{r}}=\cos\theta_Y\partial H_s(\tilde{r}-\tilde{R})/\partial\tilde{r}=\cos\theta_Y\delta(\tilde{r}-\tilde{R})$.

SI3: Finite element method

The rescaled displacement $\tilde{\boldsymbol{U}}$ is computed by solving the governing equations (6)-(8) together with the boundary conditions (9)- (14) by using a finite element method (FEM) with a Newton solver from the FEM library FEniCS.[1] Handling the delta function and the step function re-

quires careful numerical treatment to ensure adequate resolution and accuracy. To address this issue, we employ appropriate approximation techniques for both functions. For the step function $H_s(\tilde{r}-\tilde{R})$, we approximate it with the inverse of tangent function as $H_s(\tilde{r}-\tilde{R}) \approx F_s(r;R,\ell_m) = \arctan\left[(r-R)/\ell_m\right]/\pi + 1/2$, where l_m can be interpreted as a microscopic length[2–4] such that $l_m \ll l$. As the derivative of a step function is a delta function, we approximate the Dirac delta function by the derivative of $F_s(r;R,\ell_m)$. Defining $F_d(r;R,\ell_m) = \partial F_s/\partial r$, we approximate $\delta(r-R) \approx F_d(r;R,\ell_m) = l_m/\pi\left[(R-r)^2 + l_m^2\right]$. In the limit that $\ell_m \to 0$, $F_d(r;R,\ell_m \to 0) = \delta(r-R)$.[5] For all our computations, we take $\ell_m/l = 10^{-7}$.

We have used the adaptive mesh sizing such that the mesh size far away from the contact line is chosen to ensure the change of $|\theta - \theta_Y|$ is less than 2%. The smallest mesh size in the contact line region is 1% of l_m.

Mesh convergence of the numerical solver is demonstrated for the sessile droplet case shown in Fig 1 in which $\tilde{u}_z$ is plotted as a function of $\tilde{r}-\tilde{R}$ with three mesh resolutions: $\tilde{H}/d\tilde{x} = 20$, 40 and 80, where $d\tilde{x}$ is the mesh size far away from the contact line. The inset shows the zoom into the dimple position. The plot demonstrates good mesh convergence.

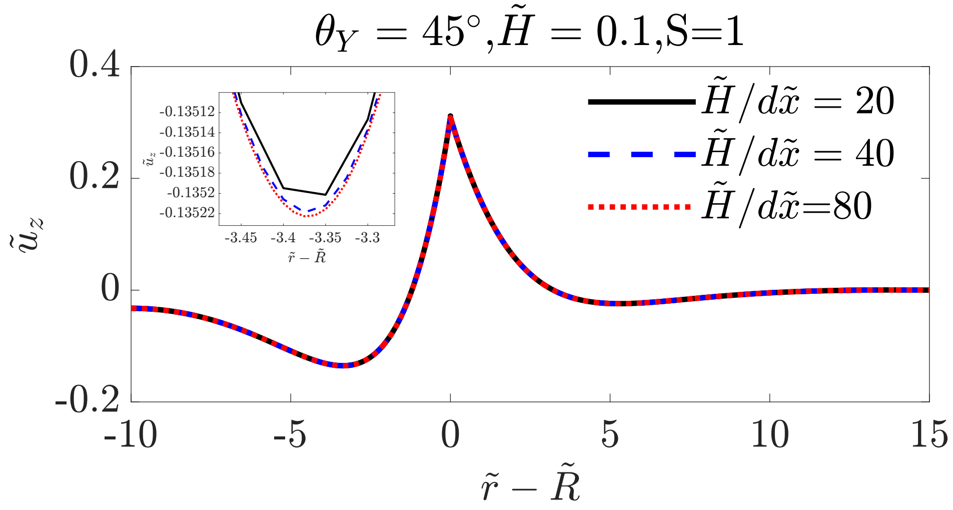

Figure 1 The rescaled displacement $\tilde{u}_z$ as a function of $\tilde{r}-\tilde{R}$ for 3 different mesh size $\tilde{H}/d\tilde{x}_0$ is the mesh size far away from the contact line.We show the numerical results with 3 different mesh sizes.The inset: the zoom into the dimple position.

References

(1) Logg, A.; Mardal, K.-A.; Wells, G. *Automated solution of differential equations by the finite element method: The FEniCS book*; Springer Science & Business Media, 2012; Vol. 84.

(2) Hui, C. Y.; Jagota, A. Deformation near a liquid contact line on an elastic substrate. *Proceedings of the Royal Society A: Mathematical, Physical and Engineering Sciences* **2014**, *470*.

(3) Dervaux, J.; Limat, L. Deformation near a liquid contact line on an elastic substrate. *Proceedings of the Royal Society A: Mathematical, Physical and Engineering Sciences* **2015**, *471*.

(4) Chan, T. S. The growth and the decay of a visco-elastocapillary ridge by localized forces. *Soft Matter* **2022**, 7280–7290.

(5) Karpitschka, S.; Das, S.; Van Gorcum, M.; Perrin, H.; Andreotti, B.; Snoeijer, J. H. Droplets move over viscoelastic substrates by surfing a ridge. *Nature Communications* **2015**, *6*, 1–7.
