# Supplementary material for: Soft Wetting Ridge Rotation in Sessile Droplets and Capillary Bridges
Source: Langmuir. 2025 Feb 3;41(6):4146–53. doi: 10.1021/acs.langmuir.4c04667 (PMC11841037; doi:10.1021/acs.langmuir.4c04667)
Supplement: Supplementary file 2 — la4c04667_si_002.zip [file la4c04667_si_002.zip › SupportingInformation/old/SupportingInformation.pdf]

Soft wetting ridge rotation] Soft wetting ridge rotation in sessile droplets and capillary bridges (Supporting information)

Supporting Information for: Your Manuscript Title

Contents

1 SI1: Relations between the components of the stress tensor and the displacements in cylindrical coordinates 4

2 SI2: The dimensionless governing equations and boundary conditions 4

3 SI3: Finite element method 5

Contents

1 SI1: Relations between the components of the stress tensor and the displacements in cylindrical coordinates

$$\sigma_{rr} = -p + \frac{E}{(1+\nu)}\left(\frac{\partial U_r}{\partial r} - \frac{1}{3}\nabla \cdot U\right), \tag{1}$$

$$\sigma_{zz} = -p + \frac{E}{(1+\nu)}\left(\frac{\partial U_z}{\partial z} - \frac{1}{3}\nabla \cdot U\right), \tag{2}$$

$$\sigma_{\phi\phi} = -p + \frac{E}{(1+\nu)}\left(\frac{U_r}{r} - \frac{1}{3}\nabla \cdot U\right), \tag{3}$$

$$\sigma_{rz} = \frac{E}{(1+\nu)}\left(\frac{\partial U_r}{\partial z} + \frac{\partial U_z}{\partial r}\right), \tag{4}$$

where the isotropic part of the stress tensor (or the pressure)

$$p = -\frac{E}{3(1-2\nu)}\nabla \cdot U. \tag{5}$$

2 SI2: The dimensionless governing equations and boundary conditions

For the elastic deformation, the dimensionless form of $\bigtriangledown \cdot \sigma = 0$ is

$$\tilde{\nabla}^2 \tilde{U}_r - \frac{\tilde{U}_r}{\tilde{r}^2} - \frac{\partial \tilde{p}}{\partial \tilde{r}} = 0 \tag{6}$$

in r-direction and

$$\tilde{\nabla}^2 \tilde{U}_z - \frac{\partial \tilde{p}}{\partial \tilde{z}} = 0 \tag{7}$$

in z-direction. The dimensionless form of the incompressibility condition (eq. **??**) is

$$\tilde{\nabla} \cdot \tilde{U} = 0. \tag{8}$$

The boundary conditions far away from the droplet at $\tilde{r} = \tilde{L}$ and at the soft/rigid solid interface respectively are

$$\tilde{U}(\tilde{r} = \tilde{L}, \tilde{z}) = 0 \tag{9}$$

and

$$\tilde{U}(\tilde{r}, \tilde{z} = 0) = 0. \tag{10}$$

At $\tilde{r} = 0$, the symmetry property gives

$$\tilde{U_r}(\tilde{r} = 0, \tilde{z}) = 0, \tag{11}$$

and

$$\frac{\partial \tilde{U_z}}{\partial \tilde{r}}(\tilde{r} = 0, \tilde{z}) = 0. \tag{12}$$

At the soft solid/fluid interface $\tilde{z} = \tilde{H}$, the force balance condition (eq. **??**) for the r-components and z-components respectively gives

$$\begin{aligned} \frac{\tilde{\sigma}_{rz}}{S} + \tilde{\kappa}_l H_s(\tilde{R} - \tilde{r}) \frac{\sin\varphi}{|\cos\varphi|} - \cos\theta\delta(\tilde{r} - \tilde{R}) \\ -\tilde{\gamma}_s \tilde{\kappa}_s \frac{\sin\varphi}{|\cos\varphi|} \\ +\frac{\partial \tilde{\gamma}_s}{\partial \tilde{r}} \cos\varphi = 0. \end{aligned} \tag{13}$$

and

$$\begin{aligned} -\frac{\tilde{\sigma}_{zz}}{S} - \tilde{\kappa}_l H_s(\tilde{R} - \tilde{r})(\cos\varphi) + \sin\theta\delta(\tilde{r} - \tilde{R}) \\ +\tilde{\gamma}_s \tilde{\kappa}_s(\cos\varphi) \\ +\frac{\partial \tilde{\gamma}_s}{\partial \tilde{r}} \sin\varphi = 0 \end{aligned} \tag{14}$$

where $\tilde{\kappa}_l = \kappa_l l$, $\tilde{\kappa}_s = \kappa_s l$, $\tilde{\gamma}_s = \frac{\gamma_s}{\gamma}$ and $\frac{\partial \tilde{\gamma}_s}{\partial \tilde{r}} = \cos\theta_Y \partial H_s(\tilde{r} - \tilde{R})/\partial \tilde{r} = \cos\theta_Y \delta(\tilde{r} - \tilde{R})$.

3 SI3: Finite element method

The rescaled displacement $\tilde{U}$ is computed by solving the governing equations (6)-(8) together with the boundary conditions (9)- (14) by using a finite element method (FEM) with a Newton solver from the FEM library FEniCS[**?**]. Handling the delta function and the step function requires careful numerical treatment to ensure adequate resolution and accuracy. To address this issue, we employ appropriate approximation techniques for both functions. For the step function $H_s(\tilde{r} - \tilde{R})$, we approximate it with the inverse of tangent function as $H_s(\tilde{r} - \tilde{R}) \approx F_s(r; R, \ell_m) = \arctan\left[(r - R)/\ell_m\right]/\pi + 1/2$, where l_m can be interpreted as a microscopic length Hui2014,Dervaux2015,Chan2022 such that $l_m \ll l$. As the derivative of a step function is a delta function, we approximate the Dirac delta function by the derivative of $F_s(r; R, \ell_m)$. Defining $F_d(r; R, \ell_m) = \partial F_s/\partial r$, we approximate $\delta(r - R) \approx F_d(r; R, \ell_m) = l_m/\pi\left[(R - r)^2 + l_m^2\right]$. In the limit that $\ell_m \to 0$, $F_d(r; R, \ell_m \to 0) = \delta(r - R)$ [**?**]. For all our computations, we take $\ell_m/l = 10^{-7}$.

[width=0.8]Figures//fig9.pdf

Figure 1: The rescaled displacement $\tilde{u}_z$ as a function of $\tilde{r} - \tilde{R}$ for 3 different mesh size $\tilde{H}/d\tilde{x}_0$ is the mesh size far away from the contact line.We show the numerical results with 3 different mesh sizes.The inset: the zoom into the dimple position.

We have used the adaptive mesh sizing such that the mesh size far away from the contact line is chosen to ensure the change of $|\theta - \theta_Y|$ is less than 2%. The smallest mesh size in the contact line region is 1% of l_m.

Mesh convergence of the numerical solver is demonstrated for the sessile droplet case shown in Fig 1 in which $\tilde{u}_z$ is plotted as a function of $\tilde{r} - \tilde{R}$ with three mesh resolutions: $\tilde{H}/d\tilde{x} = 20$, 40 and 80, where $d\tilde{x}$ is the mesh size far away from the contact line. The inset shows the zoom into the dimple position. The plot demonstrates good mesh convergence.
