# Supplementary figures and images for: Soft Wetting Ridge Rotation in Sessile Droplets and Capillary Bridges
Source: Langmuir. 2025 Feb 3;41(6):4146–53. doi: 10.1021/acs.langmuir.4c04667 (PMC11841037; doi:10.1021/acs.langmuir.4c04667)

# Capillary bridge

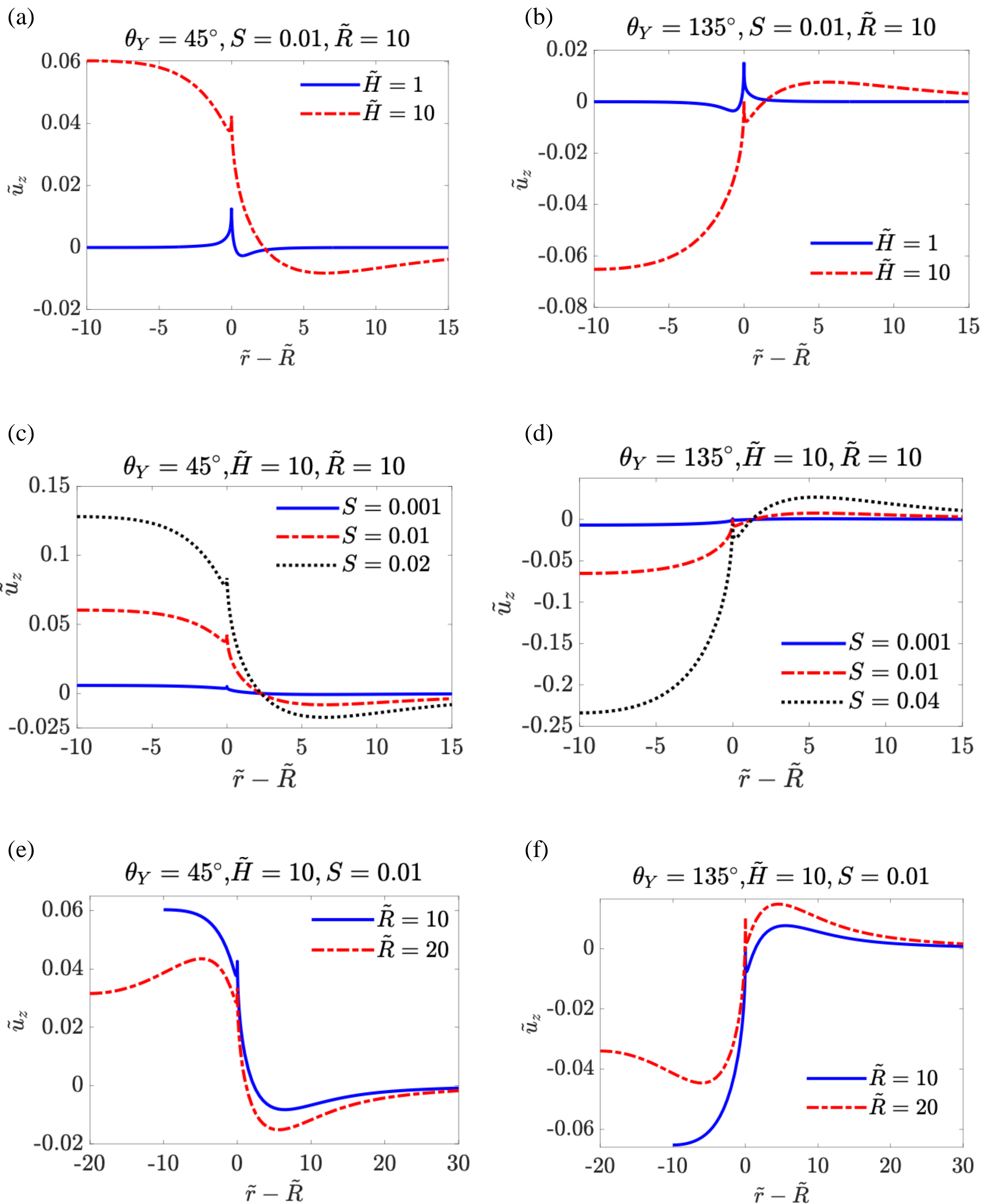

Supplement: Supplementary file 2 — la4c04667_si_002.zip [file la4c04667_si_002.zip › SupportingInformation/Figures/fig4.pdf]

# Sessile droplet

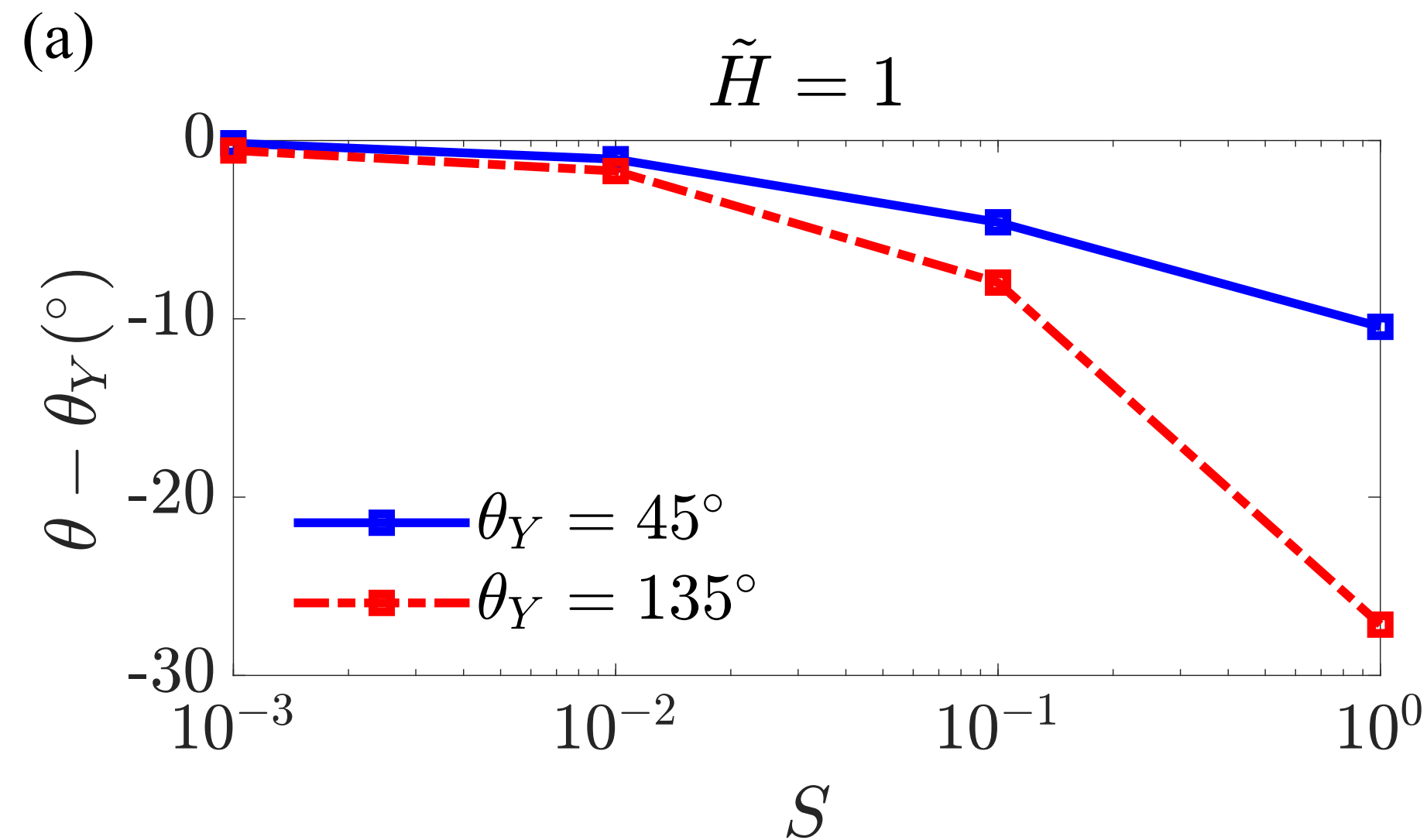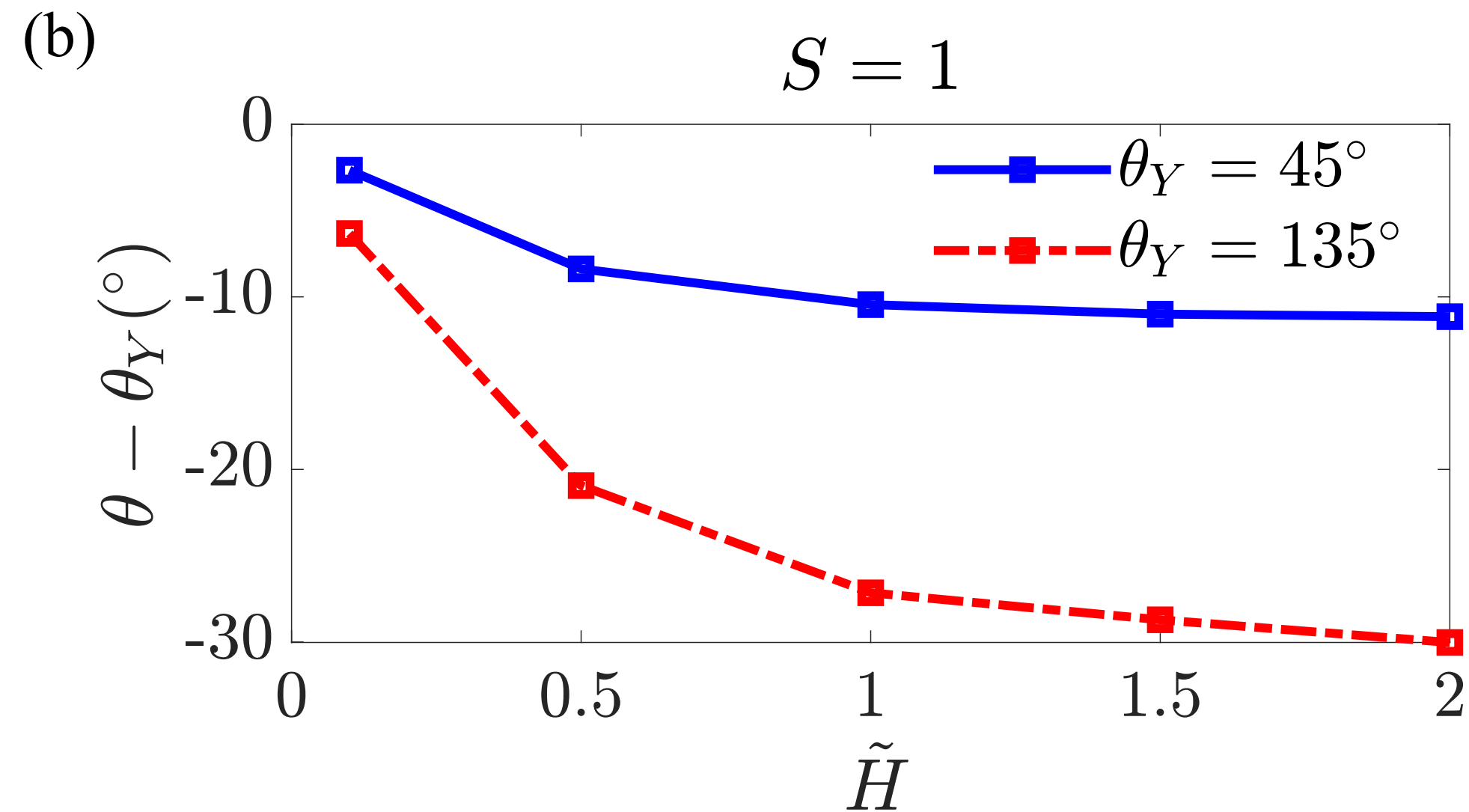

Supplement: Supplementary file 2 — la4c04667_si_002.zip [file la4c04667_si_002.zip › SupportingInformation/Figures/fig5.pdf]

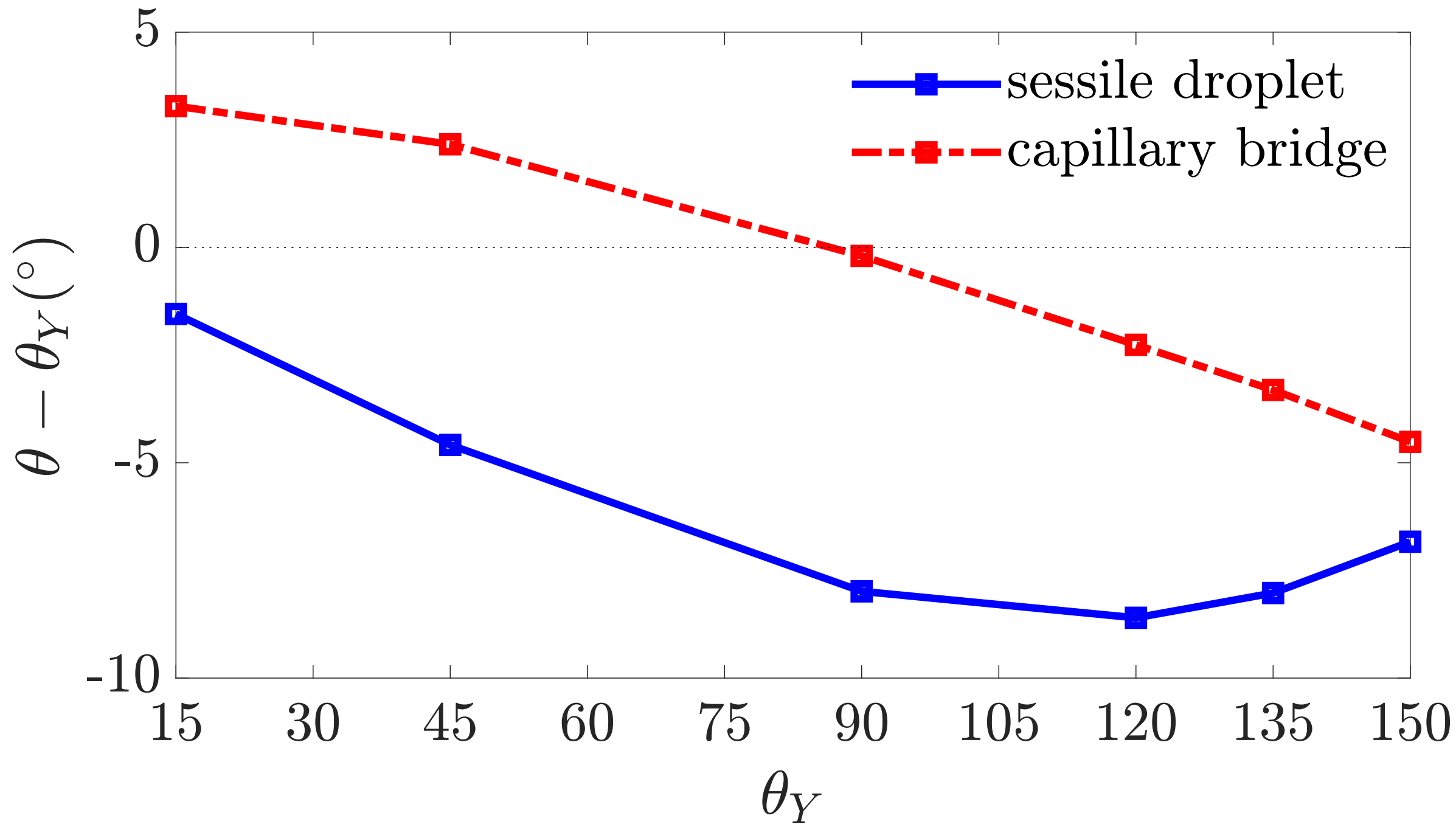

Supplement: Supplementary file 2 — la4c04667_si_002.zip [file la4c04667_si_002.zip › SupportingInformation/Figures/fig7.pdf]

# Capillary bridge

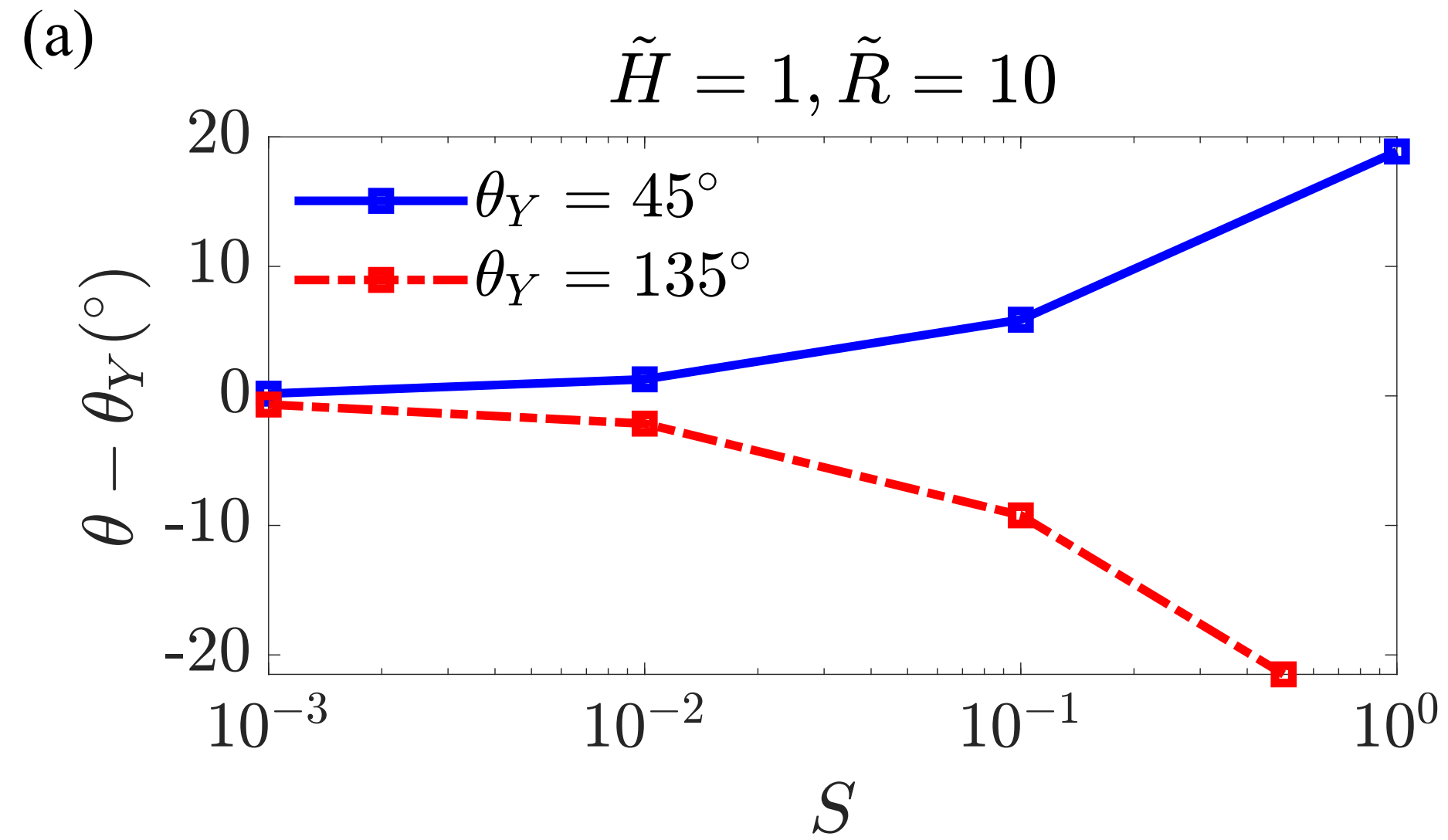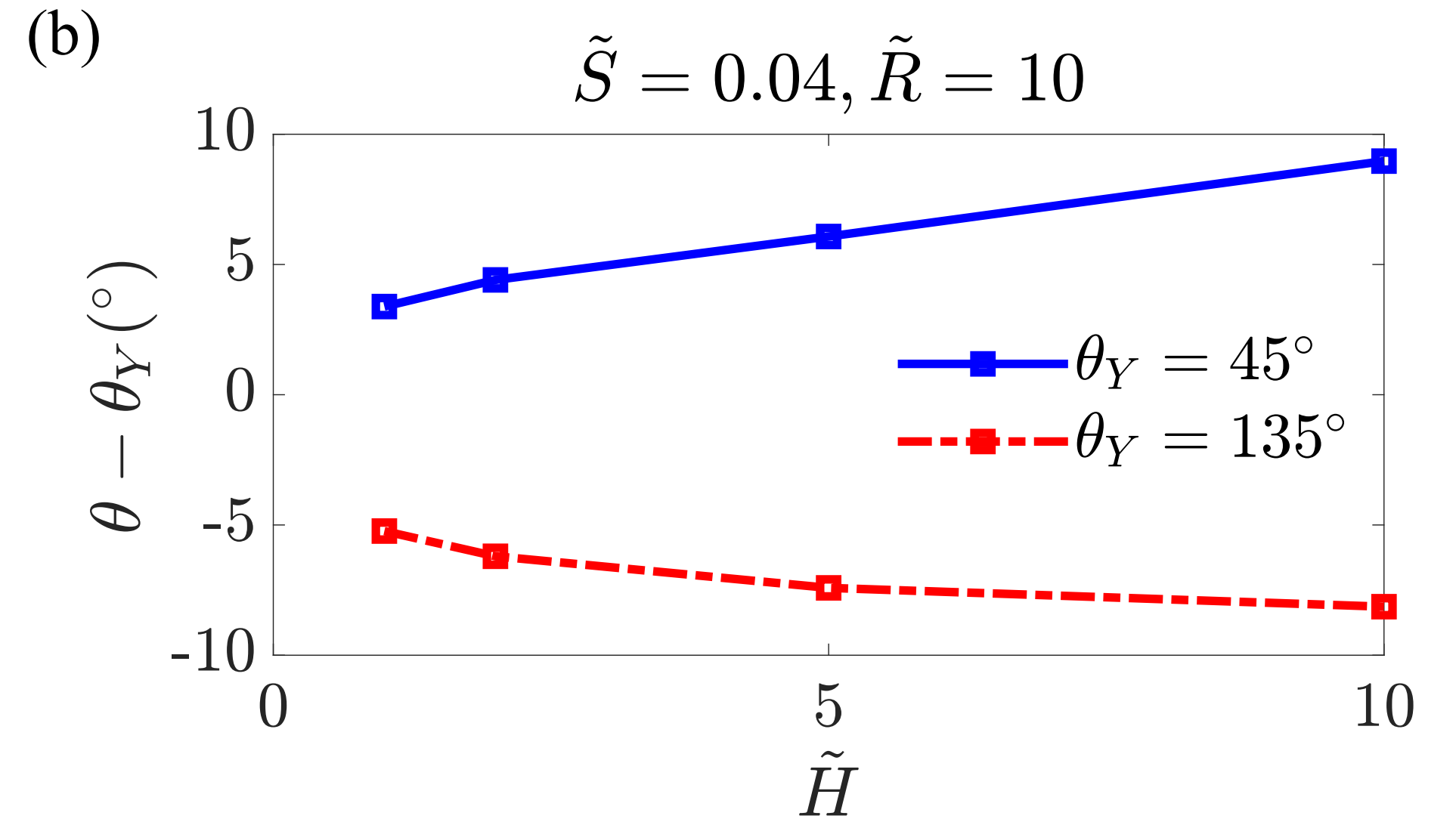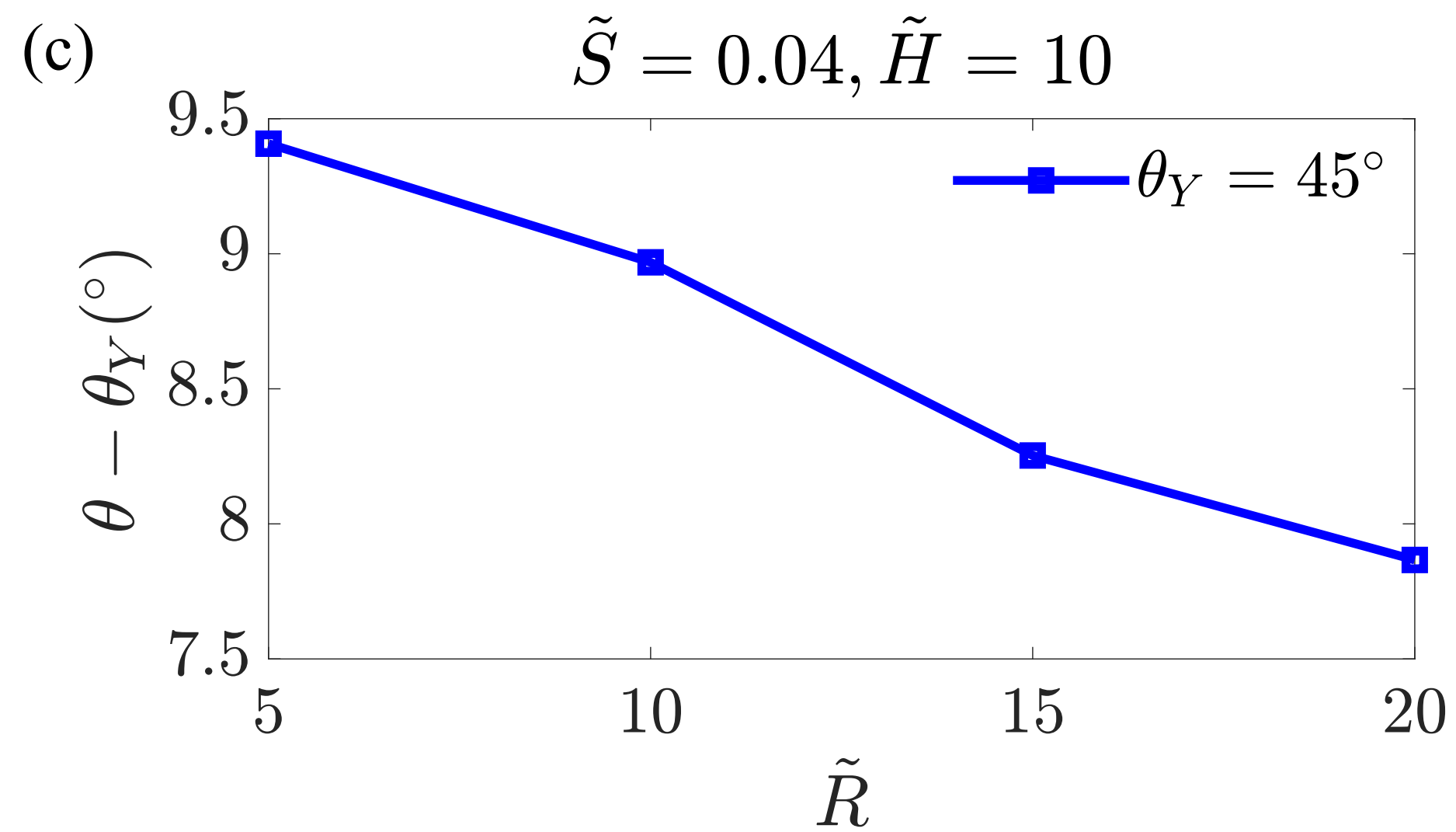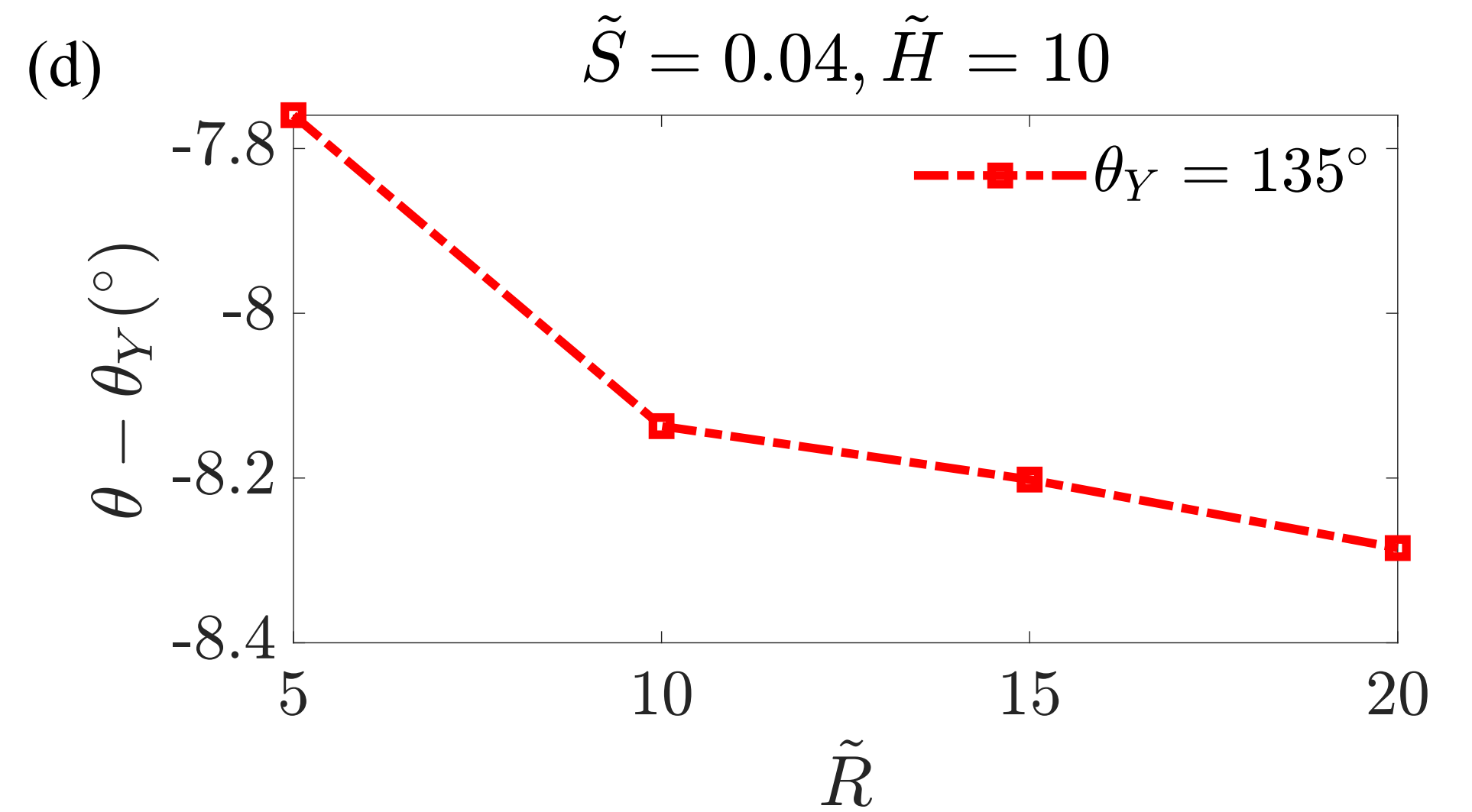

Supplement: Supplementary file 2 — la4c04667_si_002.zip [file la4c04667_si_002.zip › SupportingInformation/Figures/fig6.pdf]

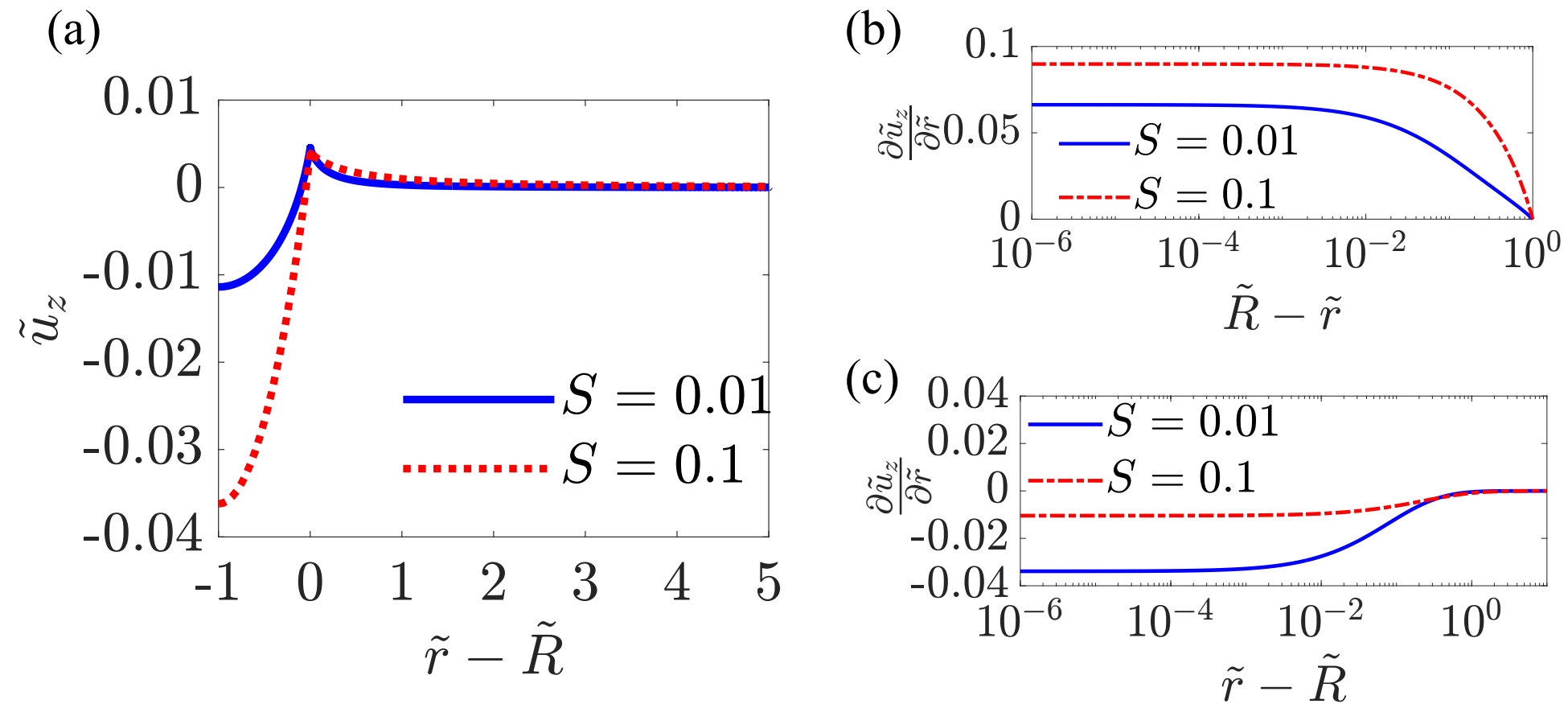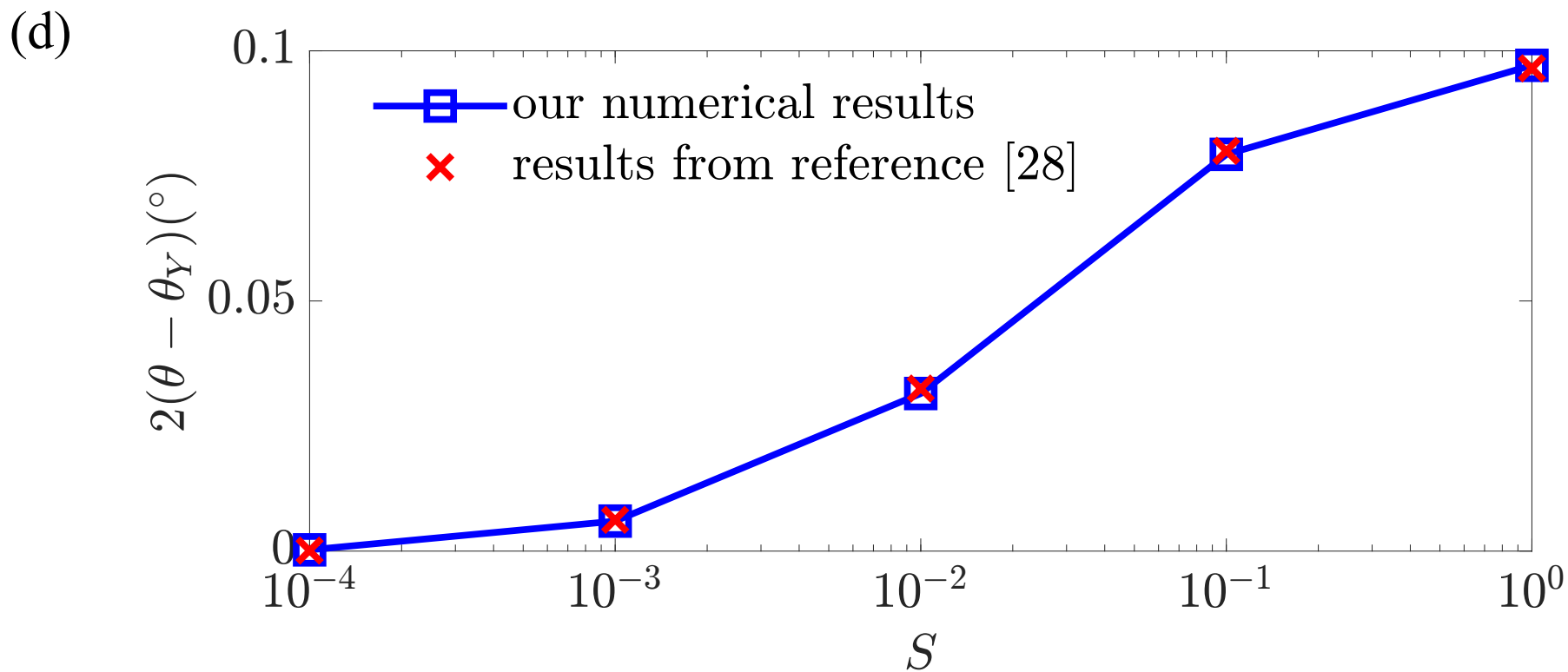

Supplement: Supplementary file 2 — la4c04667_si_002.zip [file la4c04667_si_002.zip › SupportingInformation/Figures/fig2.pdf]

# Sessile droplet

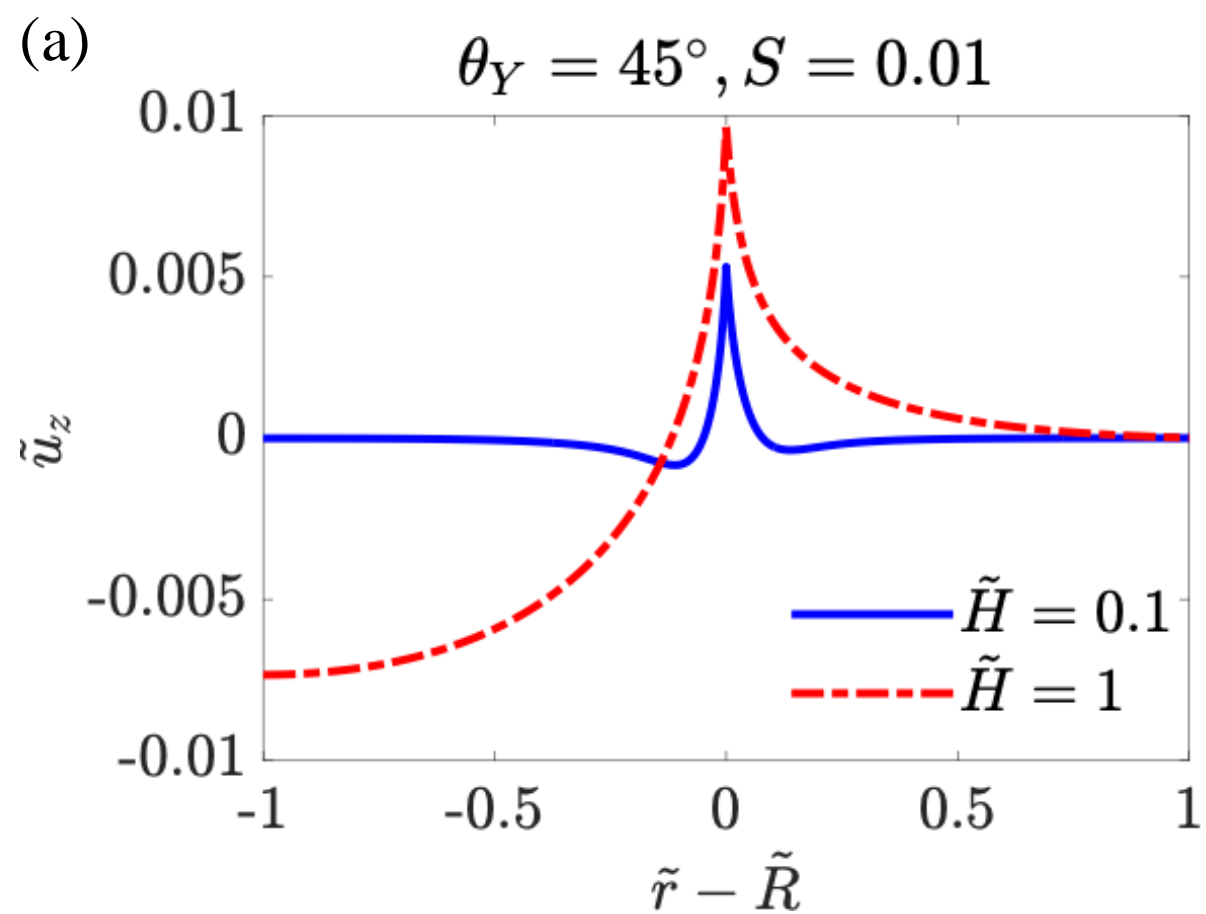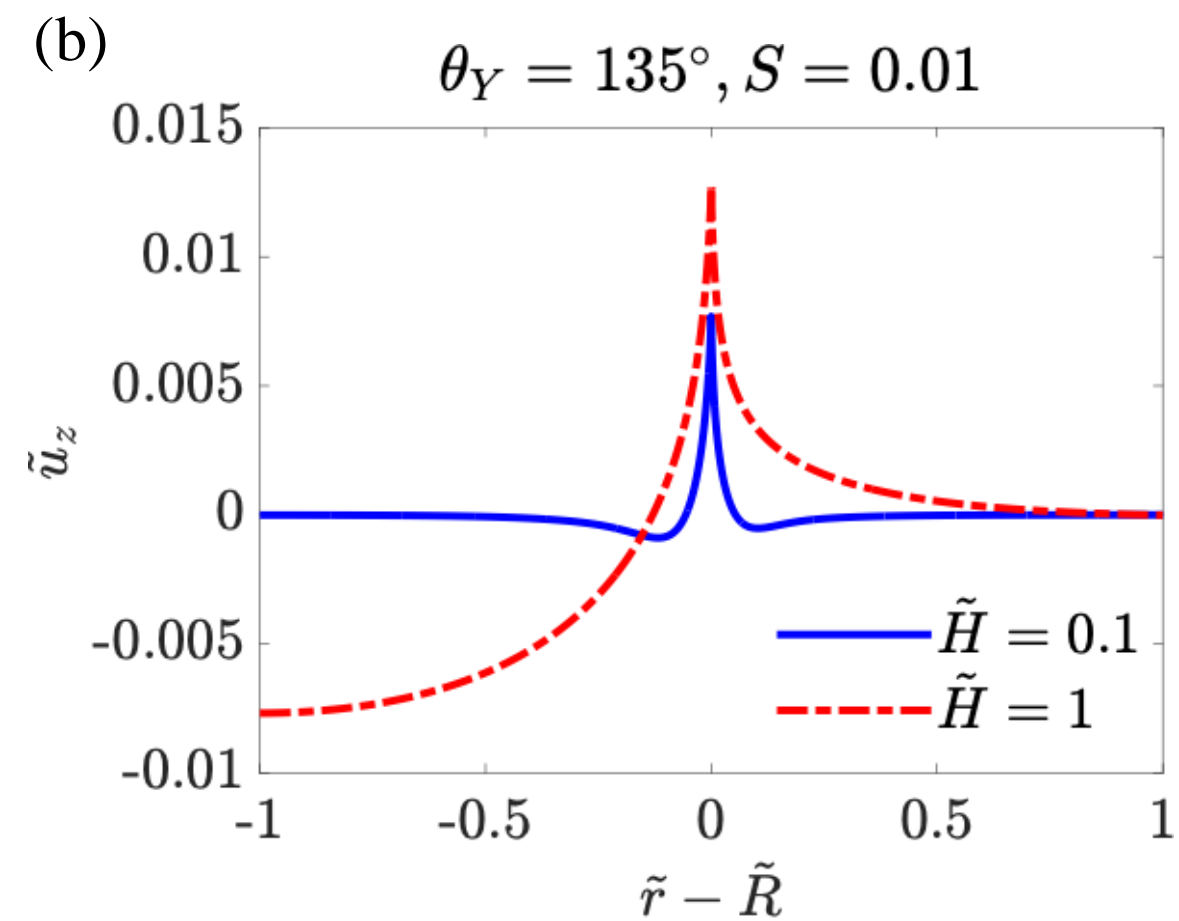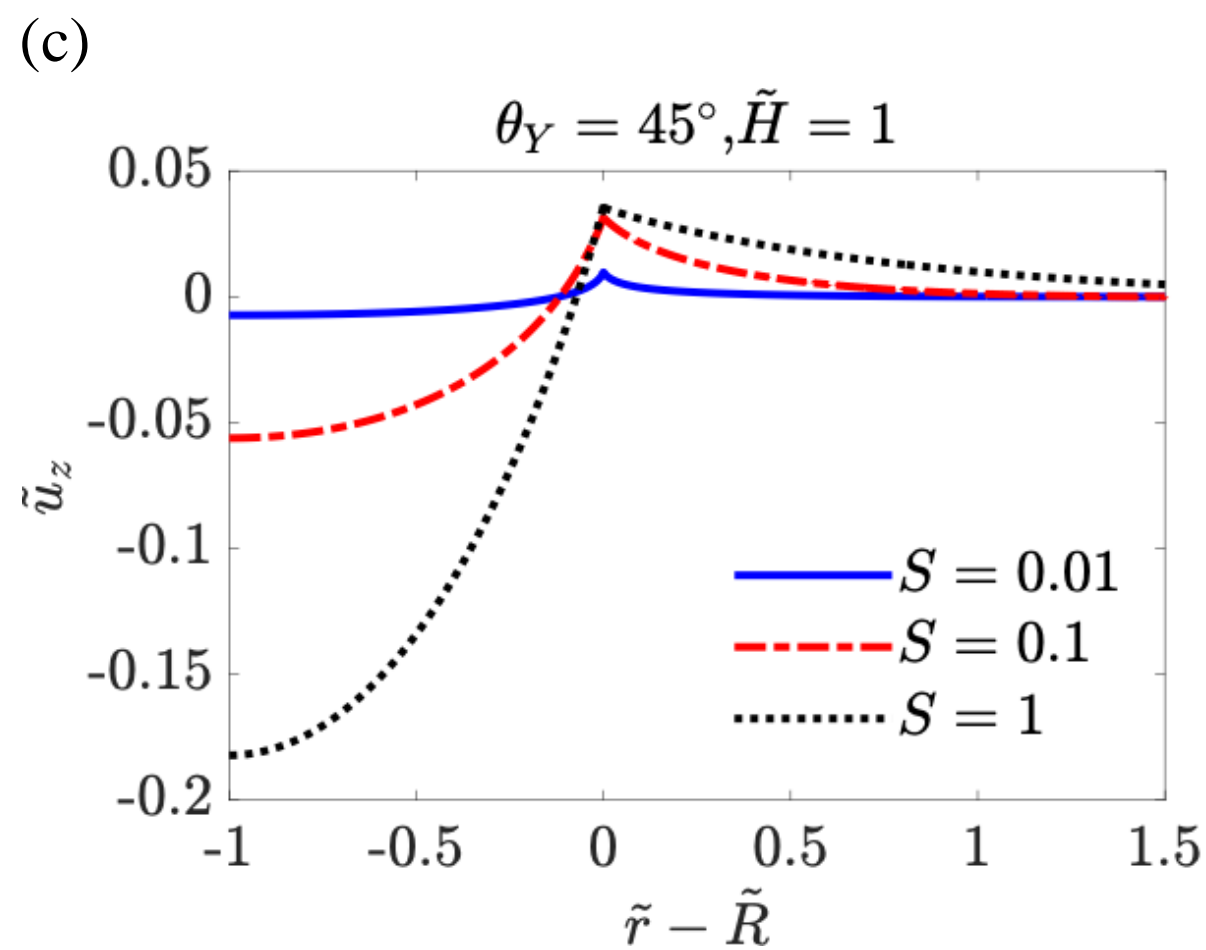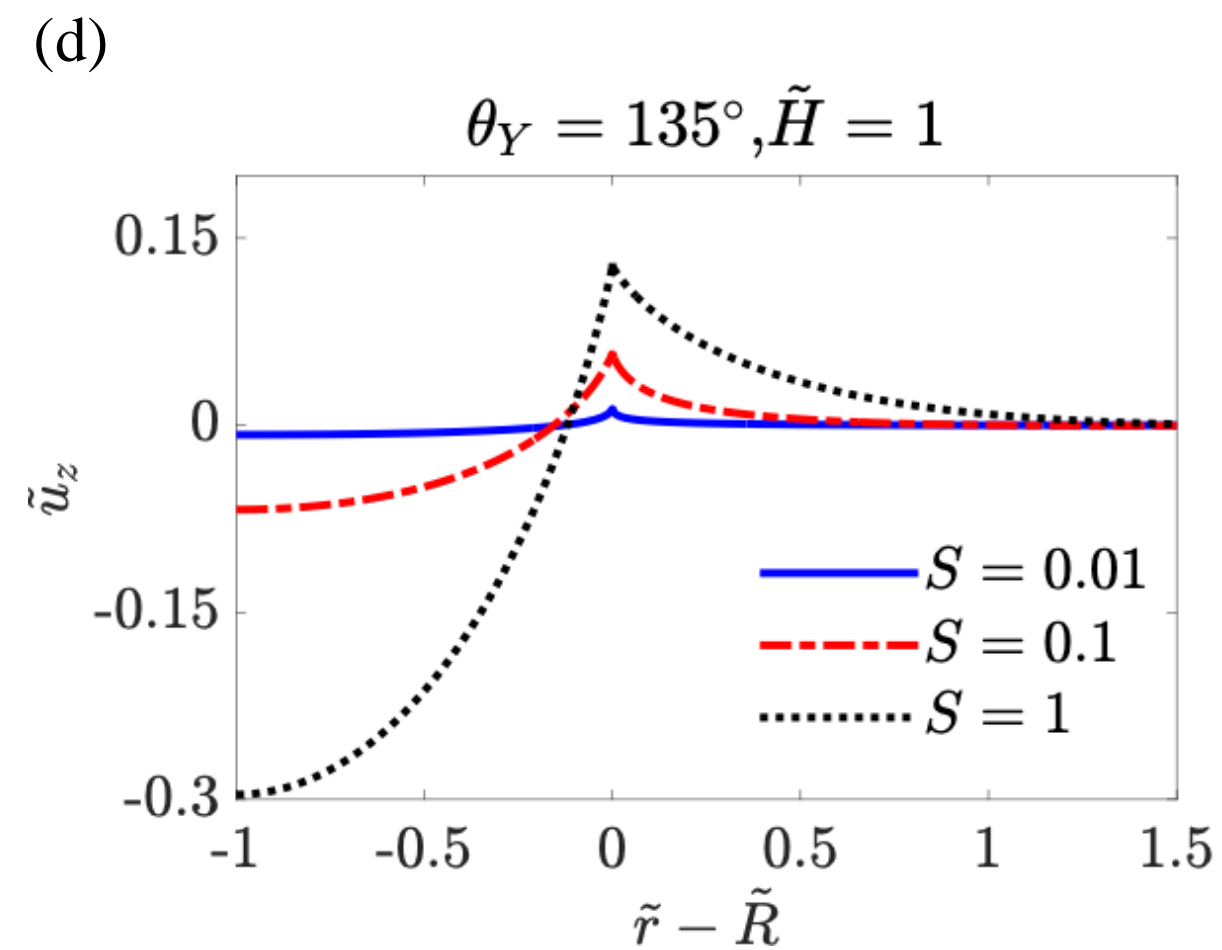

Supplement: Supplementary file 2 — la4c04667_si_002.zip [file la4c04667_si_002.zip › SupportingInformation/Figures/fig3.pdf]

(a)

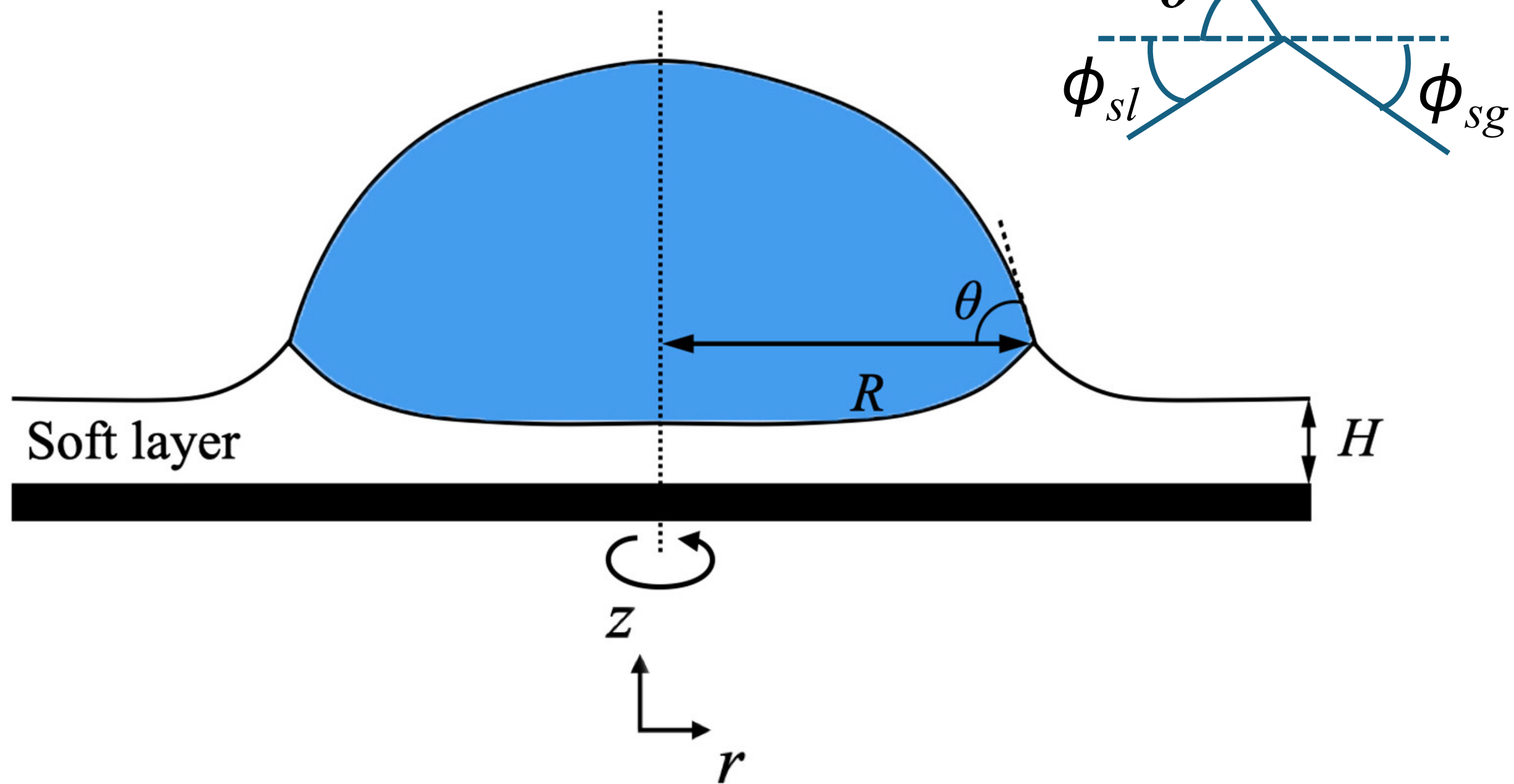

(b)

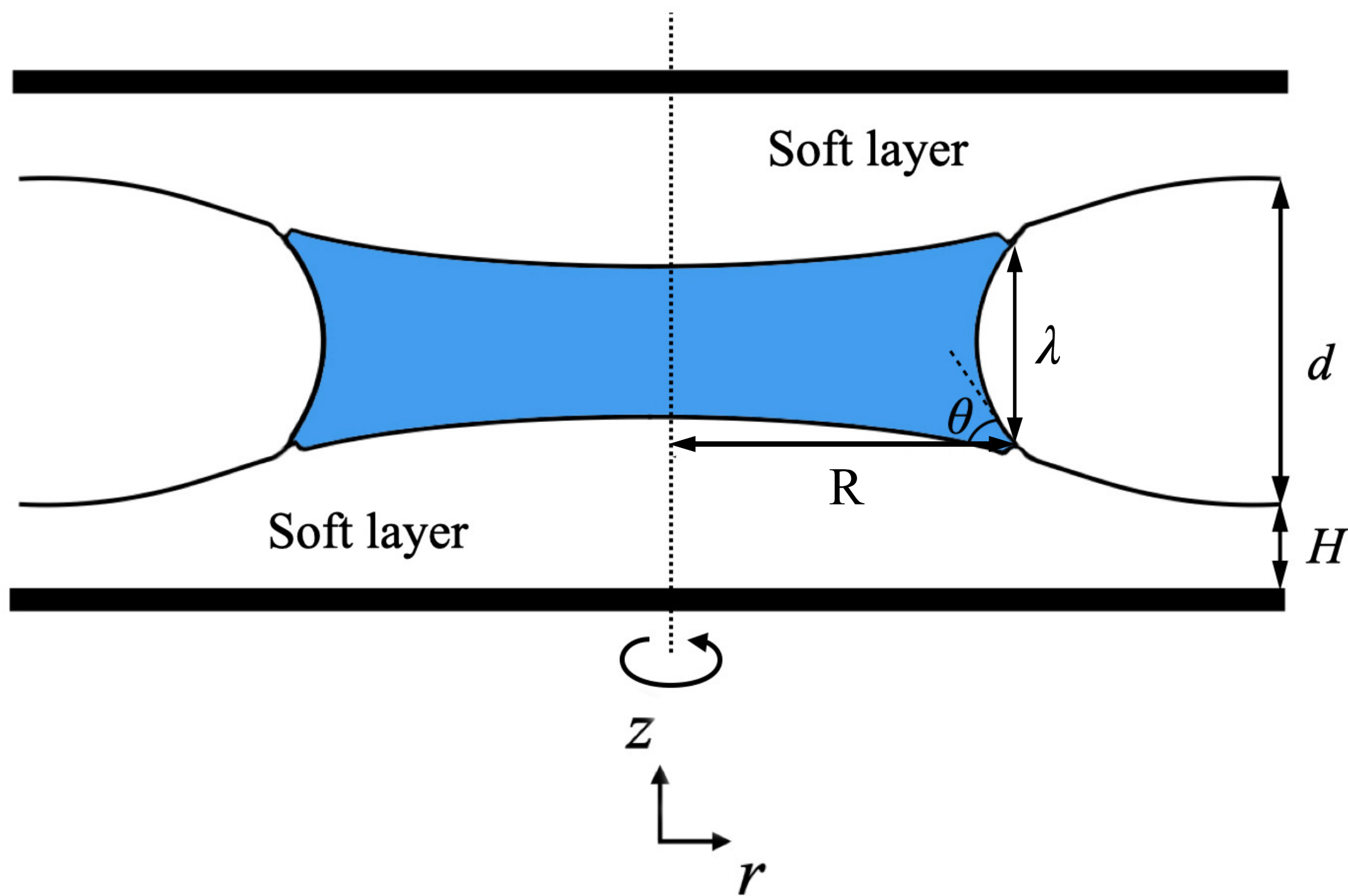

Supplement: Supplementary file 2 — la4c04667_si_002.zip [file la4c04667_si_002.zip › SupportingInformation/Figures/fig1.pdf]

# Sessile droplet

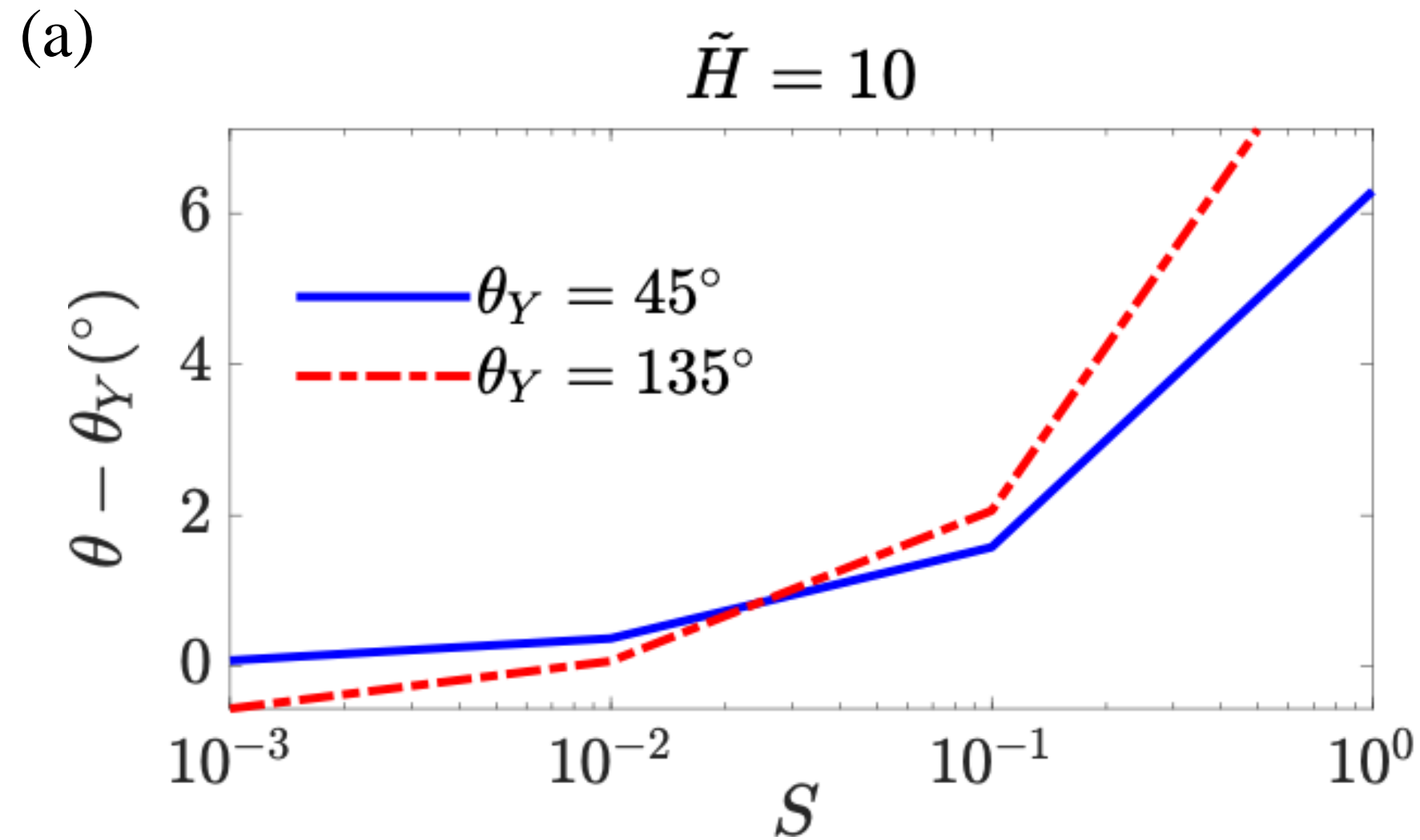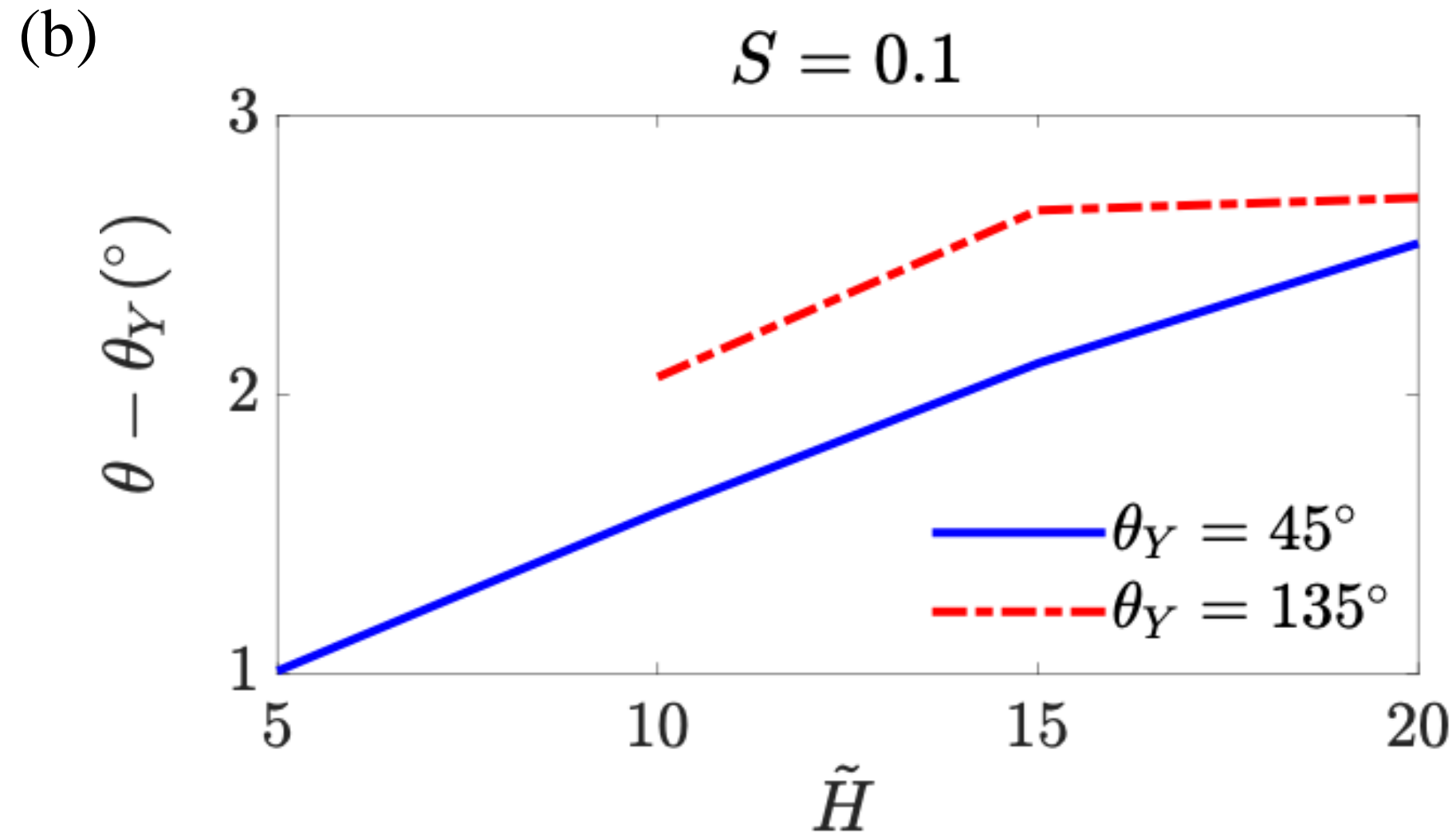

Supplement: Supplementary file 2 — la4c04667_si_002.zip [file la4c04667_si_002.zip › SupportingInformation/Figures/fig8.pdf]

$$\theta_Y = 45^\circ, \tilde{H} = 0.1, S=1$$

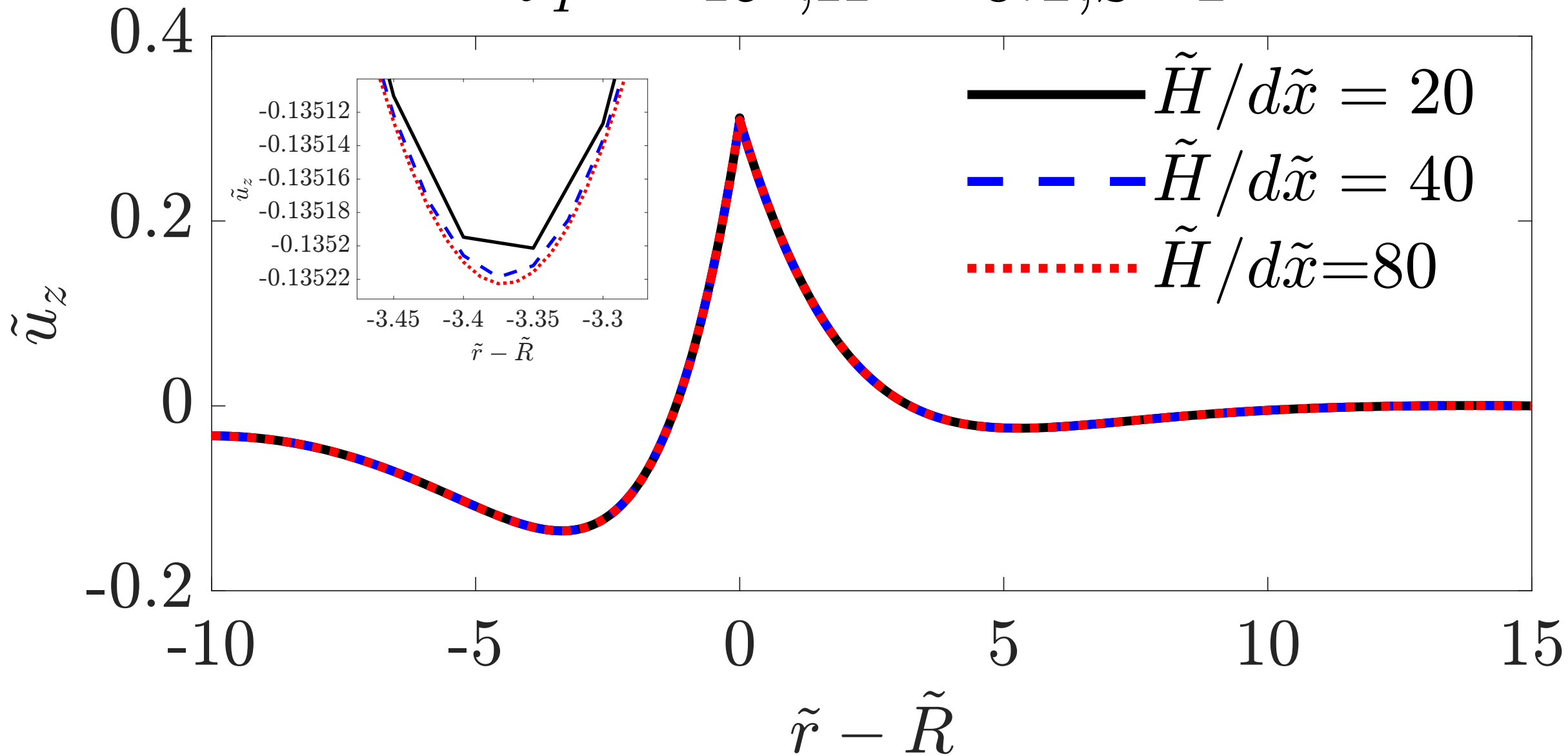

Supplement: Supplementary file 2 — la4c04667_si_002.zip [file la4c04667_si_002.zip › SupportingInformation/Figures/fig9.pdf]
